# Supplementary material for: Pharmacological inhibition of cystine–glutamate exchange induces endoplasmic reticulum stress and ferroptosis
Source: eLife. 2014 May 20;3:e02523. doi: 10.7554/eLife.02523 (PMC4054777; doi:10.7554/eLife.02523)
Supplement: Supplementary file 1. — Extended materials and methods. Description of chemical synthesis and characterization. Data also available as ‘Extended Materials and Methods’ from Dryad data Repository: http://dx.doi.org/10.5061/dryad.jp43c. DOI: http://dx.doi.org/10.7554/eLife.02523.016 [file elife02523s001.docx]

**Extended Materials and Methods**

**Chemistry**

A. Total synthesis of erastin and analogs

**General information**. All reactions were carried out under a nitrogen atmosphere under anhydrous conditions unless indicated otherwise. Anhydrous methylene chloride (DCM), tetrahydrofuran (THF), N,N-dimethylformamide (DMF) was purchased from Sigma-Aldrich. Reactions were magnetically stirred and monitored by thin layer chromatography carried out by Merck pre-coated 0.25 mm silica plates containing a 254 nm fluorescence indicator. Flash chromatography was preformed on a Teledyne Combiflash companion automatic flash chromatography system. Preparative thin layer chromatography was performed on 1 mm. Spectroscopy: NMR spectra were obtained on a Bruker DPX 300 or 400 MHz spectrometer.

## Abbreviations. EDIPA = diisopropylethyl amine, EtOAc = ethyl acetate, Et2O = diethyl ether, MeOH = methanol, EtOH = ethanol, Pd(PPh_3_)_4_ = Tetrakis(triphenylphosphine)palladium(0), Na_2_SO_4_ = sodium sulfate, MgSO_4_ =

## DMAP = 4-dimethylaminopyridine, POCl_3_ = phosphorous oxychloride, NaHCO_3_ = sodium bicarbonate, TBS-Cl = *tert*-butylchlorodimethylsilane, NBS = N-bromosuccinimide, NaBH_4_ = sodium borohydride, NH_4_Cl = ammonium chloride, TFA = triflouroacetic acid, PBr_3_ = phosphours tribromide, HBTU = O-(Benzotriazol-1-yl)-N,N,N′,N′-tetramethyluronium hexafluorophosphate

**Quinoline Erastin (scheme 2) – 6MEW78**

**N-phenylethanamide.** Acetyl chloride (0.916 mL, 12.89 mmol, 1.2 eq) was added slowly to a solution of EDIPA (2.25 mL, 12.89 mmol, 1.2 eq) and aniline (0.98 mL, 10.74 mmol) at 0 °C. The resulting mixture was stirred for an additional hour at 0 °C then at 25 °C for 4 hours. Upon completion, the mixture was quenched with saturated aqueous NaHCO_3_ and extracted three times with EtOAc. The combined organic layers were dried (Na_2_SO_4_), concentrated, and the crude material was purified by combiflash 0-> 20% EtOAc in hexanes to provide N-phenylethanamide (1.27 g, 88% yield).

**2-chloroquinoline-3-carbaldehyde**. POCl_3_ (1.29 mL, 14.1 mmol, 9.5 eq) was added to DMF (0.379 mL, 4.89 mmol, 3.3 eq) at 0 °C. The mixture was stirred for an additional 5 min at 0 °C before the addition of N-phenylethanamide (200 mg, 1.48 mmol). The mixture was then heated to 75 °C and stirred for 12 hours. Upon completion, the reaction was quenched carefully with cold water and extracted three times with Et_2_O. The combined organic layers were dried (MgSO_4_), concentrated, and the crude material was purified by combiflash 0->30% EtOAc in hexanes to provide 2-chloroquinoline-3-carbaldehyde (139 mg, 49% yield).

**(2-chloroquinolin-3-yl)methanol**. NaBH_4_ (0.849 g, 22.44 mmol, 2.0 eq) was added to a solution of 2-chloroquinoline-3-carbaldehyde (2.15 g, 11.22 mmol) in DCM:MeOH (2:1, 120 mL) at 0° C and stirred for 1 hour. Upon completion, the reaction was quenched with saturated aqueous NH_4_Cl and extracted three times with dichloromethane. The combined organic layers were dried (Na_2_SO_4_), concentrated, and the crude material was purified by combiflash 0->50% EtOAc in hexanes to provide (2-chloroquinolin-3-yl)methanol (2.1 g, 97%).

**3-((*tert*-butyldimethylsilyloxy)methyl)-2-chloroquinoline** was prepared according to the general TBS protection procedure (1.2 g, 29%)

**3-((*tert*-butyldimethylsilyloxy)methyl)-2-(2-isopropoxyphenyl)quinoline** (*Suzuki coupling procedure*). Pd(PPh_3_)_4_ (187 mg, 0.162 mmol, 10%) was added to a solution of 3-((*tert*-butyldimethylsilyloxy)methyl)-2-chloroquinoline (0.5 g, 1.62 mmol) in dioxane (10 mL) and stirred for 10 min at 25° C. To the resulting mixture, a solution of 2-isopropoxyphenylboronic acid (0.438 g, 2.43 mmol, 1.5 eq) in EtOH (4 mL) was added and the resulting mixture was stirred at 25 °C for 10 min before the addition of a 2 M aqueous solution of sodium carbonate (8.1 mL, 16.2 mmol, 10 eq). The resulting mixture was stirred at 25 °C for an additional 5 min, then heated to 80 °C for 24 hours. Upon completion, the mixture was diluted with water and extracted three times with EtOAc. The combined organic layers were dried (Na_2_SO_4_), concentrated, and the crude material was purified by combiflash 0->20% EtOAc in hexanes to provide 3-((*tert*-butyldimethylsilyloxy)methyl)-2-(2-isopropoxyphenyl)quinoline (0.57 g, 86% yield)

**(2-(2-isopropoxyphenyl)quinolin-3-yl)methanol**. TBAF (1 M, 0.67 mL, 0.67 mmol, 1.1 eq) was added to a solution of (2-(2-isopropoxyphenyl)quinolin-3-yl)methanol in THF (6 mL) at 0 °C. The resulting mixture was stirred at 0 °C for 1 hour. Upon completion, saturated aqueous NH_4_Cl was added and extracted 3 times with EtOAc. The combined organic layers were dried (Na_2_SO_4_), concentrated, and the crude material was purified by combiflash 0->40% EtOAc in hexanes to provide (2-(2-isopropoxyphenyl)quinolin-3-yl)methanol (135 mg, 75%).

**3-(bromomethyl)-2-(2-isopropoxyphenyl)quinolone**. PBr_3_ (0.130 mL, 1.38 mmol, 3 eq) was added to a solution of (2-(2-isopropoxyphenyl)quinolin-3-yl)methanol (135 mg, 0.45 mmol) in DCM (5 mL) at 0 °C. The resulting mixture was stirred at 0 °C for an additional 30 min, then at 25 °C for 2 hr. Upon completion, the reaction was quenched with saturated aqueous NaHCO_3_ and extracted 3 times with EtOAc. The combined organic layers were dried (Na_2_SO_4_), concentrated, and the crude material was purified by combiflash 0-20% EtOAc in hexanes to provide 3-(bromomethyl)-2-(2-isopropoxyphenyl)quinoline (57 mg, 35%).

**2-(2-isopropoxyphenyl)-3-(piperazin-1-ylmethyl)quinoline (amine addition procedure)**. To a solution of 3-(bromomethyl)-2-(2-isopropoxyphenyl)quinoline (57 mg, 0.160 mmol) in THF, 2 mL at 0 °C, piperzine (138 mg, 1.6 mmol, 10 eq) was added and stirred for 12 hours. The reaction mixture was then concentrated and purified directly by combiflash 0-20% MeOH in DCM to afford 2-(2-isopropoxyphenyl)-3-(piperazin-1-ylmethyl)quinoline (51 mg, 88% yield).

**6MEW78** **- 2-(2-isopropoxyphenyl)-3-(piperazin-1-ylmethyl)quinoline** (acyl chloride addition procedure). To a solution of 2-(2-isopropoxyphenyl)-3-(piperazin-1-ylmethyl)quinoline (51 mg, 0.141 mmol), EDIPA (30 uL, 0.169 mmol, 1.2 eq) and 4-DMAP (3 mg, 0.0282 mmol, 0.2 eq) in DCM (2 mL) at 0 °C, a solution of 4-chlorophenoxy acetyl chloride (32 mg, 0.155 mmol, 1.1 eq) in DCM (1 mL) was added dropwise. The reaction slowly warmed to 25 °C and was stirred for 4 hours. Upon completion, the reaction was quenched with saturated aqueous NaHCO_3_ and extracted three times with DCM. The combined organic layers were dried (Na_2_SO_4_), concentrated, and the crude material was purified by preparative TLC 5% MeOH in DCM (34 mg, 45% yield) ^1^H NMR (300 MHz, chloroform-d) δ 8.29 p.p.m. (s, 1H), 8.23-8.04 (m, 1H), 7.88 (dd, J = 8.0, 1.5 Hz, 1H), 7.71 (ddd, J = 8.4, 6.9, 1.5 Hz, 1H), 7.57 (ddd, J = 8.1, 6.9, 1.2 Hz, 1H), 7.47-7.31 (m, 2H), 7.27-7.19 (m, 2H), 7.09 (td, J = 7.5, 7.4, 1.0 Hz, 1H), 6.99 (d, J = 8.3 Hz, 1H), 6.91 ? 6.82 (m, 2H), 4.64 (s, 2H), 4.43 (p, J = 6.0, 6.0, 6.0, 6.0 Hz, 1H), 3.85-3.37 (m, 6H), 2.34 (m, 4H), 1.22 (d, J = 6.0 Hz, 3H), 1.03 (d, J = 6.0 Hz, 3H). HRMS (m/z): [M+] cald for C31H32ClN3O3 530.06, found 530.22.

**7MEW81 - 2-((*tert*-butyldimethylsilyloxy)methyl)-1H-indole** (TBS protection procedure). TBS-Cl (1.66 g, 11.04 mmol, 1.2 eq) was added to a solution of (1H-indol-2-yl)methanol (1.335 g, 9.2 mmol) and EDIPA (2.4 mL, 13.8 mmol, 1.5 eq) in DMF (30 mL) at 25 °C. The mixture was stirred for an additional 5 hours at 25 °C. Upon completion, the reaction contents were diluted with water and extracted three times with Et_2_O. The combined organic layers were dried (MgSO_4_), concentrated, and the crude material was purified by combiflash 0-20% EtOAc in hexanes to provide 2-((tert-butyldimethylsilyloxy)methyl)-1H-indole (2.04 g, 85%)

**3-bromo-2-((*tert*-butyldimethylsilyloxy)methyl)-1H-indole**. NBS (0.34 g, 1.91 mmol) was added to a stirred solution of 2-((tert-butyldimethylsilyloxy)methyl)-1H-indole (0.5 g, 1.91 mmol) in DMF at 0 °C. The mixture was stirred an additional 1 hour at 0 °C. Upon completion, the reaction contents were diluted with water and extracted 3 times with Et_2_O. The combined organic layers were dried (MgSO_4_), concentrated, and the crude material was purified by combiflash 0->10% EtOAc in hexanes to provide 3-bromo-2-((*tert*-butyldimethylsilyloxy)methyl)-1H-indole (0.514 g, 79%)

***Tert*-butyl 3-bromo-2-((tert-butyldimethylsilyloxy)methyl)-1H-indole-1-carboxylate**. Di-*tert*-butyl dicarbonate (2.08 g, 9.52 mmol,1.8 eq) and 4-DMAP (0.129 g, 1.06 mmol, 0.2 eq) were added sequentially to a stirred solution of 3-bromo-2-((*tert*-butyldimethylsilyloxy)methyl)-1H-indole (1.8 g, 5.29 mmol) in DCM (20 mL) at 25 °C. The mixture was stirred for an additional 12 hours. Upon completion, the reaction contents were diluted with DCM, poured into water, and extracted 2 times with DCM. The crude material was pushed forward to the next step without additional purification (1.72 g, 74% yield)

*tert*-butyl 2-((tert-butyldimethylsilyloxy)methyl)-3-(2-isopropoxyphenyl)-1H-indole-1-carboxylate was prepared according to general Suzuki coupling procedure (0.883 g, 79%).

***Tert*-butyl 2-(hydroxymethyl)-3-(2-isopropoxyphenyl)-1H-indole-1-carboxylate**. Hydrogen fluoride pyridine (0.66 mL, 70% as HF, 30% as py) was added to a solution of *tert*-butyl 2-((tert-butyldimethylsilyloxy)methyl)-3-(2-isopropoxyphenyl)-1H-indole-1-carboxylate (0.3 g, 1.01 mmol) in THF:pyridine (5:1, 10 mL) at 0 °C. The resulting mixture was then brought to room temperature and stirred for an additional 6 hours. Upon completion, the reaction was quenched with saturated NH_4_Cl and extracted three times with EtOAc. The combined organic layers were dried (Na_2_SO_4_), concentrated, and the crude material was purified by combiflash 0-30% EtOAc in hexanes to provide *tert*-butyl 2-(hydroxymethyl)-3-(2-isopropoxyphenyl)-1H-indole-1-carboxylate (0.279 g, 73%).

***Tert*-butyl 2-formyl-3-(2-isopropoxyphenyl)-1H-indole-1-carboxylate**. Dess-Martin periodinane (230 mg, 0.543 mmol, 1.8 eq) was added to a stirred suspension of sodium bicarbonate (242 mg, 2.88 mmol, 10.0 eq) and *tert*-butyl 2-(hydroxymethyl)-3-(2-isopropoxyphenyl)-1H-indole-1-carboxylate (110 mg, 0.288 mmol) in DCM (3 mL). The mixture was stirred at 25 °C for 2 hours, and upon completion, a saturated aqueous solution of sodium sulfite (1 mL) was added and the mixture was stirred for an additional 5 min. This was followed subsequently by the addition of water and extraction with DCM three times. The combined organic layers were dried (Na_2_SO_4_), concentrated, and the crude material was purified by combiflash 0-40% EtOAc in hexanes to give tert-butyl 2-formyl-3-(2-isopropoxyphenyl)-1H-indole-1-carboxylate (quantitative yield).

***Tert*-butyl 3-(2-isopropoxyphenyl)-2-(piperazin-1-ylmethyl)-1H-indole-1-carboxylate**. A solution of *tert*-butyl 2-formyl-3-(2-isopropoxyphenyl)-1H-indole-1-carboxylate (0.140 g, 0.369 mmol) in DCM (1.5 mL)was added to a stirred solution of piperazine (0.318 g, 3.69 mmol, 10 eq) in MeOH (1.5 mL) at 25 °C. After an additional 15 min of stirring a solution of ZnCl_2_ (10 mg, 0.0738 mmol, 0.2 eq) and sodium cyanoborohydride (70 mg, 1.11 mmol, 3.0 eq) in MeOH (1.5 eq) was added. The resulting mixture was heated to 40 °C and stirred for 6 hours. Upon completion, the reaction was diluted with water and extracted three times with EtOAc. The combined organic layers were dried (Na_2_SO_4_), concentrated, and the crude material was purified by combiflash 0->20% MeOH in DCM to provide tert-butyl 3-(2-isopropoxyphenyl)-2-(piperazin-1-ylmethyl)-1H-indole-1-carboxylate (73 mg, 44% yield).

***Tert*-butyl 2-((4-(2-(4-chlorophenoxy)ethanoyl)piperazin-1-yl)methyl)-3-(2-isopropoxyphenyl)-1H-indole-1-carboxylate** was prepared according to the general acyl chloride addition procedure (89 mg, 90%).

**7MEW81- 2-(4-chlorophenoxy)-1-(4-((3-(2-isopropoxyphenyl)-1H-indol-2-yl)methyl)piperazin-1-yl)ethanone**. TFA (1 mL) was added to a solution of *tert*-butyl 2-((4-(2-(4-chlorophenoxy)ethanoyl)piperazin-1-yl)methyl)-3-(2-isopropoxyphenyl)-1H-indole-1-carboxylate (85 mg, 0.138 mmol) in DCM (1 mL) and the resulting solution was stirred at 25 °C for 24 hours. Upon completion, the reaction was quenched with saturated aqueous NaHCO_3_ and extracted three times with EtOAc. The combined organic layers were dried (Na_2_SO_4_), concentrated, and the crude material was purified by combiflash 0->5% MeOH in DCM to provide 2-(4-chlorophenoxy)-1-(4-((3-(2-isopropoxyphenyl)-1H-indol-2-yl)methyl)piperazin-1-yl)ethanone (50 mg, 70% yield). ^1^H NMR (300 MHz, chloroform-d) δ 8.61 p.p.m. (s, 1H), 7.52 (d, J = 7.9 Hz, 1H), 7.40 (ddd, J = 7.3, 5.8, 1.5 Hz, 2H), 7.39-7.27 (m, 1H), 7.30-7.15 (m, 3H), 7.17-6.99 (m, 3H), 6.94-6.81 (m, 2H), 4.66 (s, 2H), 4.35 (p, J = 6.1, 6.1, 6.1, 6.1 Hz, 1H), 3.93-3.40 (m, 7H), 2.43 (d, J = 9.7 Hz, 4H), 1.24-1.00 (m, 6H). HRMS (m/z): [M+] cald for C30H32ClN3O3 518.05, found 517.21.

Procedures for forming the quinzaolinone core (the synthesis of compound 2, scheme 1 from anthranillic acid derivatives) are described in Yang et al (*submitted*).

**2-((4-(2-chloroethanoyl)piperazin-1-yl)methyl)-3-(2-isopropoxyphenyl)quinazolin-4(3H)-one**. To a solution of a solution of 3-(2-isopropoxyphenyl)-2-(piperazin-1-ylmethyl)quinazolin-4(3H)-one (200 mg, 0.53 mmol) and EDIPA (82 uL, 0.635 mmol, 1.2 eq) in DCM (5 mL) at 0 °C, a solution of chloroacetyl chloride (50 uL, 0.635 mmol, 1.2 eq) was added and the resulting mixture was stirred at 25 °C for 4 hours. Upon completion, the reaction was quenched with saturated aqueous NaHCO_3_ and extracted three times with DCM. The combined organic layers were dried (Na_2_SO_4_), concentrated, and the crude material was purified by combiflash 0->5% MeOH in DCM to provide 2-((4-(2-chloroethanoyl)piperazin-1-yl)methyl)-3-(2-isopropoxyphenyl)quinazolin-4(3H)-one (210 mg, 73% yield)

**35MEW22 - 2-((4-(2-(4-chlorophenylamino)ethanoyl)piperazin-1-yl)methyl)-3-(2-isopropoxyphenyl)quinazolin-4(3H)-one**. 4-chloroaniline (29 mg, 0.230 mmol, 5.0 eq) was added to a solution of 2-((4-(2-chloroethanoyl)piperazin-1-yl)methyl)-3-(2-isopropoxyphenyl)quinazolin-4(3H)-one (21 mg, 0.0462 mmol) in DMF (0.5 mL) and the resulting mixture was stirred at 60 °C for 12 hours. Upon completion, the mixture was diluted with water and extracted three times with diethyl ether. The combined organic layers were dried (MgSO_4_), concentrated, and the crude material was purified by preparative TLC 5% MeOH in DCM to provide 2-((4-(2-(4-chlorophenylamino)ethanoyl)piperazin-1-yl)methyl)-3-(2-isopropoxyphenyl)quinazolin-4(3H)-one (8 mg, 32% yield). ^1^H NMR (400 MHz, Chloroform-d) δ 8.32 p.p.m. (dq, J = 8.0, 1.6, 1.6, 1.5 Hz, 1H), 7.87-7.71 (m, 2H), 7.64-7.39 (m, 2H), 7.31 (s, 1H), 7.19-6.96 (m, 5H), 6.56-6.46 (m, 2H), 4.91 (d, J = 6.0 Hz, 1H), 4.57 (dtd, J = 8.1, 6.3, 5.8, 4.2 Hz, 1H), 3.80 (dt, J = 3.9, 1.8, 1.8 Hz, 2H), 3.58 (d, J = 5.6 Hz, 2H), 3.41-3.26 (m, 4H), 2.66- 2.43 (m, 2H), 2.28 (dt, J = 16.2, 7.1, 7.1 Hz, 2H), 1.26 (dt, J = 6.1, 1.8, 1.8 Hz, 4H), 1.17 (dt, J = 6.2, 1.7, 1.7 Hz, 3H). HRMS (m/z): [M+] cald for C30H32ClN5O3 546.06, found 546.23

3-(2-isopropoxyphenyl)-2-((methyl(2(methylamino)ethyl)amino)methyl)quinazolin-4(3H)-one was prepared according to the general amine addition procedure using N,N-dimethylethylene diamine in place of piperazine (27 mg, 56% yield).

**35MEW26** was prepared from 3-(2-isopropoxyphenyl)-2-((methyl(2(methylamino)ethyl)amino)methyl)quinazolin-4(3H)-one (20 mg, 22% yield). ^1^H NMR (400 MHz, chloroform-d) δ 8.36-8.26 p.p.m. (m, 1H), 7.81-7.70 (m, 2H), 7.54-7.39 (m, 2H), 7.27-7.16 (m, 3H), 7.09 (td, J = 7.9, 7.4, 4.1 Hz, 2H), 6.92-6.80 (m, 2H), 4.73 (s, 1H), 4.61 (s, 1H), 4.57 (ddt, J = 9.2, 6.1, 3.1, 3.1 Hz, 1H), 3.49-3.30 (m, 4H), 3.01 (s, 1H), 2.90 (s, 2H), 2.82-2.44 (m, 2H), 2.29 (d, J = 15.6 Hz, 3H), 1.25 (d, J = 3.7 Hz, 3H), 1.16 (dd, J = 6.3, 1.3 Hz, 3H). HRMS (m/z): [M+] cald for C30H33ClN4O4 549.04, found 549.22

**3-(2-isopropoxyphenyl)-2-((methylamino)methyl)quinazolin-4(3H)-one**.

A 33% (wt) solution (EtOH) of methyl amine (0.15 mL) was added to a solution of 2-(chloromethyl)-3-(2-isopropoxyphenyl)quinazolin-4(3H)-one (80 mg, 0.243 mmol) in THF (3 mL) and the resulting mixture was stirred at 25 °C for 12 hr. Upon completion, the mixture was diluted with water and extracted 3 times with EtOAc. The combined organic layers were dried (Na_2_SO_4_), concentrated, and the crude material was purified by combi flash 0->10% MeOH in DCM to provide 3-(2-isopropoxyphenyl)-2-((methylamino)methyl)quinazolin-4(3H)-one (43 mg, 55% yield).

**35MEW27** was prepared from 3-(2-isopropoxyphenyl)-2-((methylamino)methyl)quinazolin-4(3H)-one using general acyl chloride addition procedure (71 mg, 72% yield) Mixture of atropisomers ^1^H NMR (400 MHz, chloroform-d) δ 8.31 p.p.m. (d, J = 7.9 Hz, 2H), 7.80 (dddd, J = 12.6, 8.5, 7.2, 1.5 Hz, 2H), 7.64 (dd, J = 8.3, 1.1 Hz, 1H), 7.59-7.41 (m, 5H), 7.25 (dd, J = 8.2, 6.1 Hz, 3H), 7.20-7.07 (m, 6H), 7.00-6.94 (m, 2H), 6.86-6.75 (m, 2H), 4.82 (s, 2H), 4.60 (d, J = 7.6 Hz, 5H), 4.25 (s, 2H), 3.98 (d, J = 17.0 Hz, 1H), 3.22 (s, 3H), 3.06 (s, 3H), 1.30 (dd, J = 18.1, 6.1 Hz, 7H), 1.18 (dd, J = 16.3, 6.0 Hz, 6H). HRMS (m/z): [M+] cald for C27H26ClN3O4 491.97, found 492.13.

**35MEW14**. To a solution of 3-(2-isopropoxyphenyl)-2-(piperazin-1-ylmethyl)quinazolin-4(3H)-one (30 mg, 0.079 mmol) and potassium carbonate (13 mg, 0.094 mmol, 1.2 eq) in DMF (1 mL), 4-chlorophenyl 2-bromoethyl ether (28 mg, 0.118 mmol, 1.5 eq) was added. The resulting mixture was stirred at 60 °C for 24 hours. Upon completion, the mixture was diluted with saturated aqueous NaHCO_3_ and extracted three times with EtOAc. The combined organic layers were dried (Na_2_SO_4_), concentrated, and the crude material was purified preparative TLC, 10% MeOH in DCM to provide 35MEW14 (11.8 mg, 28% yield). ^1^H NMR (400 MHz, chloroform-d) δ 8.32 p.p.m. (dd, J = 7.9, 1.1 Hz, 1H), 7.88-7.75 (m, 2H), 7.50 (dt, J = 8.1, 4.1, 4.1 Hz, 1H), 7.43 (td, J = 8.1, 7.9, 1.7 Hz, 1H), 7.31 (dd, J = 7.7, 1.7 Hz, 1H), 7.27-7.20 (m, 2H), 7.12-7.00 (m, 2H), 6.90-6.76 (m, 2H), 4.56 (p, J = 6.0, 6.0, 6.0, 6.0 Hz, 1H), 4.06 (t, J = 5.8, 5.8 Hz, 2H), 3.26 (d, J = 1.4 Hz, 2H), 2.78 (t, J = 5.8, 5.8 Hz, 2H), 2.51 (s, 6H), 2.35-2.22 (m, 2H), 1.25 (d, J = 6.0 Hz, 3H), 1.17 (d, J = 6.0 Hz, 3H). HRMS (m/z): [M+] cald for C30H33ClN4O3 533.06, found 533.23.

**35MEW38** (amine coupling procedure). HBTU (147 mg, 0.38 mmol, 1.5 eq) was added to a solution of 3-chlorophenoxy acetic acid (69 mg, 0.336 mmol, 1.3 eq) and EDIPA (67 uL, 0.38 mmol, 1.5 eq) in DCM (3 mL) and stirred for 30 min at 25 °C. A solution of 3-(2-isopropoxyphenyl)-2-(piperazin-1-ylmethyl)quinazolin-4(3H)-one (100 mg, 0.258 mmol) in DCM (1 mL) was added and stirred for an additional 4 hours. Upon completion, the reaction was quenched with saturated aqueous NaHCO_3_ and extracted three times with DCM. The combined organic layers were dried (Na_2_SO_4_), concentrated, and the crude material was purified by combi flash 0->5% MeOH in DCM to provide 35MEW38 (120 mg, 85% yield). ^1^H NMR (400 MHz, chloroform-d) δ 8.38-8.27 p.p.m. (m, 1H), 7.87-7.68 (m, 2H), 7.51 (ddd, J = 8.2, 6.6, 1.7 Hz, 1H), 7.43 (ddd, J = 8.3, 7.5, 1.7 Hz, 1H), 7.33-7.26 (m, 1H), 7.21 (t, J = 8.1, 8.1 Hz, 1H), 7.12-7.04 (m, 2H), 6.97 (ddd, J = 8.0, 1.9, 0.9 Hz, 1H), 6.92 (t, J = 2.2, 2.2 Hz, 1H), 6.83 (ddd, J = 8.5, 2.5, 0.9 Hz, 1H), 4.64 (s, 2H), 4.56 (p, J = 6.1, 6.1, 6.0, 6.0 Hz, 1H), 3.68-3.36 (m, 4H), 3.29 (s, 2H), 2.49 (dddd, J = 26.4, 11.1, 6.1, 3.2 Hz, 2H), 2.32-2.12 (m, 2H), 1.24 (d, J = 6.1 Hz, 3H), 1.16 (d, J = 6.0 Hz, 3H). HRMS (m/z): [M+] cald for C36H35ClN4O4 547.04, found 547.21

**35MEW39**. Prepared using the general amine coupling procedure from 3-(2-isopropoxyphenyl)-2-(piperazin-1-ylmethyl)quinazolin-4(3H)-one, (132 mg, 96% yield). ^1^H NMR (400 MHz, chloroform-d) δ 8.36-8.25 p.p.m. (m, 1H), 7.83 ? 7.72 (m, 2H), 7.50 (ddd, J = 8.1, 6.8, 1.5 Hz, 1H), 7.47-7.38 (m, 1H), 7.29-7.22 (m, 1H), 7.11-7.02 (m, 2H), 7.01-6.91 (m, 2H), 6.91-6.80 (m, 2H), 4.62 (s, 2H), 4.56 (p, J = 6.1, 6.1, 6.1, 6.1 Hz, 1H), 3.58 -3.39 (m, 5H), 3.27 (s, 2H), 2.47 (dddd, J = 29.4, 10.8, 6.2, 3.5 Hz, 2H), 2.35-2.13 (m, 2H), 1.23 (d, J = 6.0 Hz, 3H), 1.15 (d, J = 6.0 Hz, 3H). HRMS (m/z): [M+] cald for C30H31FN4O4 530.59, found 531.24

**35MEW13**. Prepared using the general amine coupling procedure from 3-(2-isopropoxyphenyl)-2-(piperazin-1-ylmethyl)quinazolin-4(3H)-one (18 mg, 44% yield). ^1^H NMR (400 MHz, chloroform-d) δ 8.32 p.p.m. (ddd, J = 8.0, 1.4, 0.7 Hz, 1H), 7.84-7.74 (m, 2H), 7.52 (ddd, J = 8.2, 6.7, 1.7 Hz, 1H), 7.44 (ddd, J = 8.4, 7.5, 1.7 Hz, 1H), 7.31 (dd, J = 7.1, 1.9 Hz, 2H), 7.29-7.27 (m, 1H), 7.12-7.06 (m, 2H), 7.00 (td, J = 7.3, 7.3, 1.1 Hz, 1H), 6.97-6.87 (m, 2H), 4.66 (s, 2H), 4.57 (p, J = 6.1, 6.1, 6.1, 6.1 Hz, 1H), 3.52 (ddt, J = 10.6, 7.6, 3.2, 3.2 Hz, 4H), 3.28 (s, 2H), 2.56-2.40 (m, 2H), 2.32-2.15 (m, 2H), 1.25 (d, J = 6.1 Hz, 3H), 1.17 (d, J = 6.0 Hz, 3H). HRMS (m/z): [M+] cald for C30H32N4O4 512.6, found 513.25

**15MEW81**. Prepared using the general amine coupling procedure 3-(2-isopropoxyphenyl)-2-(piperazin-1-ylmethyl)quinazolin-4(3H)-one (226 mg, 58% yield).^1^H NMR (400 MHz, chloroform-d) δ 8.36-8.30 p.p.m. (m, 1H), 7.86-7.74 (m, 2H), 7.52 (ddd, J = 8.2, 6.5, 1.8 Hz, 1H), 7.47-7.38 (m, 1H), 7.29-7.22 (m, 3H), 7.18-7.11 (m, 2H), 7.11-6.98 (m, 2H), 4.57 (p, J = 6.0 Hz, 1H), 3.52 (dt, J = 9.7, 4.6 Hz, 2H), 3.28 (s, 4H), 2.98-2.89 (m, 2H), 2.56 (t, J = 7.7 Hz, 2H), 2.41 (t, J = 5.7 Hz, 2H), 2.25 -2.10 (m, 2H), 1.25 (d, J = 6.0 Hz, 3H), 1.17 (d, J = 6.0 Hz, 3H). HRMS (m/z): [M+] cald for C31H33ClN4O3 545.07, found 545.23.

**35MEW28**. Prepared using general Suzuki coupling procedure from 21MEW26 (30 mg, 77% yield). ^1^H NMR (400 MHz, chloroform-d) δ 8.38-8.32 p.p.m. (m, 1H), 7.85-7.76 (m, 2H), 7.74-7.67 (m, 2H), 7.60-7.53 (m, 4H), 7.53-7.47 (m, 2H), 7.44 (t, J = 7.6, 7.6 Hz, 2H), 7.36 (d, J = 7.1 Hz, 1H), 7.26-7.20 (m, 2H), 7.15 (d, J = 8.6 Hz, 1H), 6.87-6.78 (m, 2H), 4.68-4.62 (m, 1H), 4.60 (s, 2H), 3.63-3.23 (m, 7H), 2.49 (ddt, J = 14.5, 10.6, 4.8, 4.8 Hz, 2H), 2.23 (ddd, J = 18.4, 11.4, 5.5 Hz, 2H), 1.28 (d, J = 6.0 Hz, 3H), 1.21 (d, J = 6.1 Hz, 3H). HRMS (m/z): [M+] cald for C36H35ClN4O4 623.14, found 623.24.

**35MEW29**. Prepared using general Suzuki coupling procedure from 21MEW26 (65 mg, 54%). ^1^H NMR (300 MHz, chloroform-d) δ 8.33 p.p.m. (ddd, J = 8.0, 1.5, 0.7 Hz, 1H), 7.85-7.72 (m, 2H), 7.68 (dd, J = 1.5, 0.9 Hz, 1H), 7.57-7.48 (m, 2H), 7.47 (t, J = 1.7, 1.7 Hz, 1H), 7.42 (d, J = 2.2 Hz, 1H), 7.27-7.20 (m, 2H), 7.08 (d, J = 8.6 Hz, 1H), 6.95-6.76 (m, 2H), 6.63 (dd, J = 1.9, 0.9 Hz, 1H), 4.61 (s, 2H), 4.56 (q, J = 6.1, 6.1, 6.1 Hz, 1H), 3.51 -3.35 (m, 4H), 3.29 (d, J = 2.2 Hz, 2H), 2.48 (dt, J = 11.0, 5.1, 5.1 Hz, 2H), 2.20 (tt, J = 10.8, 10.8, 5.1, 5.1 Hz, 2H), 1.25 (d, J = 6.0 Hz, 3H), 1.18 (d, J = 6.0 Hz, 3H). HRMS (m/z): [M+] cald for C34H33ClN4O5 613.1, found 613.22.

**14MEW31**. Prepared from 5-chloro-3-(2-isopropoxyphenyl)-2-(piperazin-1-ylmethyl)quinazolin-4(3H)-one using general acyl chloride addition procedure (122 mg, 87% yield).^1^H NMR (300 MHz, chloroform-d) δ 7.70-7.59 p.p.m. (m, 2H), 7.50 (dd, J = 5.5, 3.6 Hz, 1H), 7.47-7.39 (m, 1H), 7.28-7.23 (m, 2H), 7.13-7.03 (m, 2H), 6.92-6.82 (m, 2H), 4.64 (s, 2H), 4.57 (p, J = 6.1, 6.1, 6.1, 6.1 Hz, 1H), 3.51 (d, J = 14.4 Hz, 4H), 3.25 (s, 2H), 2.48 (s, 2H), 2.25 (s, 2H), 1.26 (d, J = 6.0 Hz, 3H), 1.19 (d, J = 6.0 Hz, 3H). HRMS (m/z): [M+] cald for C30H30Cl2N4O4 581.49, found 581.17

**14MEW32**. Prepared from 8-chloro-3-(2-isopropoxyphenyl)-2-(piperazin-1-ylmethyl)quinazolin-4(3H)-one using general acyl chloride addition procedure (77 mg, 55% yield). ^1^H NMR (300 MHz, chloroform-d) δ 8.23 p.p.m. (dd, J = 8.0, 1.5 Hz, 1H), 7.87 (dd, J = 7.8, 1.5 Hz, 1H), 7.52-7.38 (m, 2H), 7.28-7.21 (m, 4H), 7.13-7.01 (m, 2H), 6.93-6.81 (m, 2H), 4.66 (s, 2H), 4.64-4.47 (m, 1H), 3.54 (s, 4H), 3.33 (d, J = 3.0 Hz, 2H), 2.64 (s, 1H), 2.54 (s, 1H), 2.44 (s, 1H), 2.33 (s, 1H), 1.26 (d, J = 6.1 Hz, 3H), 1.18 (d, J = 6.0 Hz, 3H). HRMS (m/z): [M+] cald for C30H30Cl2N4O4 581.49, found 581.17

**13MEW16**. Prepared from 3-(3-isopropoxyphenyl)-2-(piperazin-1-ylmethyl)quinazolin-4(3H)-one using general acyl chloride addition procedure (100 mg, 54%). ^1^H NMR (400 MHz, chloroform-d) δ 8.37-8.27 p.p.m. (m, 1H), 7.82 (ddd, J = 8.4, 6.9, 1.5 Hz, 1H), 7.76 (d, J = 8.4 Hz, 1H), 7.54 (ddd, J = 8.2, 7.0, 1.4 Hz, 1H), 7.43 (t, J = 8.0, 8.0 Hz, 1H), 7.28- 7.18 (m, 2H), 7.10-6.94 (m, 1H), 6.88 (dd, J = 9.3, 2.1 Hz, 4H), 4.65 (s, 2H), 4.58 (p, J = 6.1, 6.1, 6.0, 6.0 Hz, 1H), 3.53 (dt, J = 22.0, 5.0, 5.0 Hz, 4H), 3.32 (s, 2H), 2.40 (ddt, J = 27.6, 9.7, 5.0, 5.0 Hz, 4H), 1.38 (dd, J = 6.3, 1.5 Hz, 6H). HRMS (m/z): [M+] cald for C30H31ClN4O4 547.04, found 547.21.

**13MEW76**. Prepared from 3-(2-isopropoxyphenyl)-2-(piperazin-1-ylmethyl)quinazolin-4(3H)-one using general acyl chloride addition procedure (270 mg, 62% yield). ^1^H NMR (300 MHz, chloroform-d) δ 8.32 p.p.m. (dd, J = 8.0, 1.4 Hz, 1H), 7.87-7.71 (m, 2H), 7.57-7.40 (m, 2H), 7.28-7.21 (m, 3H), 7.13-7.00 (m, 2H), 6.91-6.84 (m, 2H), 4.64 (s, 2H), 4.61-4.48 (m, 1H), 3.50 (s, 4H), 3.28 (s, 2H), 2.44 (s, 2H), 2.23 (s, 2H), 1.24 (d, J = 6.0 Hz, 3H), 1.16 (d, J = 6.0 Hz, 3H). HRMS (m/z): [M+] cald for C30H31ClN4O4 547.04, found 547.21.

**21MEW26**. Prepared from 3-(5-bromo-2-isopropoxyphenyl)-2-(piperazin-1-ylmethyl)quinazolin-4(3H)-one (1.87 g, 70% yield). ^1^H NMR (400 MHz, chloroform-d) δ 8.36-8.26 p.p.m. (m, 1H), 7.81-7.70 (m, 2H), 7.54-7.39 (m, 2H), 7.27-7.16 (m, 3H), 7.09 (td, J = 7.9, 7.4, 4.1 Hz, 2H), 6.92-6.80 (m, 2H), 4.73 (s, 1H), 4.61 (s, 1H), 4.57 (ddt, J = 9.2, 6.1, 3.1, 3.1 Hz, 1H), 3.49-3.30 (m, 4H), 3.01 (s, 1H), 2.90 (s, 2H), 2.82-2.44 (m, 2H), 2.29 (d, J = 15.6 Hz, 3H), 1.25 (d, J = 3.7 Hz, 3H), 1.16 (dd, J = 6.3, 1.3 Hz, 3H). HRMS (m/z): [M+] cald for C30H33ClN4O4 549.04, found 549.22

**10MEW79**. Prepared from 6-fluoro-3-(2-isopropoxyphenyl)-2-(piperazin-1-ylmethyl)quinazolin-4(3H)-one using general acyl chloride addition procedure (0.296 mg, 69% yield). ^1^H NMR (400 MHz, chloroform-d) δ 7.95 p.p.m. (dd, J = 8.4, 3.0 Hz, 1H), 7.76 (dd, J = 9.0, 4.8 Hz, 1H), 7.52 (td, J = 8.6, 8.5, 2.8 Hz, 1H), 7.48-7.41 (m, 1H), 7.28-7.17 (m, 3H), 7.16-7.02 (m, 2H), 6.91-6.81 (m, 2H), 4.64 (s, 2H), 4.57 (p, J = 6.1, 6.1, 6.0, 6.0 Hz, 1H), 3.61-3.39 (m, 4H), 3.27 (s, 2H), 2.46 (dq, J = 22.1, 7.0, 7.0, 4.7 Hz, 2H), 2.24 (ddt, J = 30.1, 10.7, 4.3, 4.3 Hz, 2H), 1.25 (d, J = 6.1 Hz, 3H), 1.17 (d, J = 6.0 Hz, 3H). HRMS (m/z): [M+] cald for C30H30ClFN4O4 565.04, found 565.2.

**8MEW98**. Prepared from 3-(2-isopropoxyphenyl)-6,7-dimethoxy-2-(piperazin-1-ylmethyl)quinazolin-4(3H)-one using the general acyl chloride addition procedure (54 mg, 53% yield). ^1^H NMR (300 MHz, chloroform-d) δ 7.64 p.p.m. (s, 1H), 7.50 ? 7.39 (m, 1H), 7.28 ? 7.16 (m, 5H), 7.13-7.02 (m, 2H), 6.92 ? 6.82 (m, 2H), 4.64 (s, 2H), 4.57 (q, J = 6.1, 6.1, 6.0 Hz, 1H), 4.03 (d, J = 9.2 Hz, 5H), 3.50 (d, J = 17.6 Hz, 4H), 3.24 (d, J = 1.8 Hz, 2H), 2.46 (s, 2H), 2.20 (s, 2H), 1.24 (d, J = 6.1 Hz, 3H), 1.17 (d, J = 6.0 Hz, 3H). HRMS (m/z): [M+] cald for C32H35ClN4O6 607.1, found 607.23.

**6MEW160**. To a solution of 2-((4-(2-(4-chlorophenoxy)ethanoyl)piperazin-1-yl)methyl)-3-(2-isopropoxyphenyl)-6-nitroquinazolin-4(3H)-one (60 mg, 0.1 mmol) in methanol (1 mL) 10% palladium on carbon (3 mg) was added. The reaction was then stirred under hydrogen gas (1 atm) for 12 hr. Upon completion the mixture was filtered over celite, concentrated, and purified by combiflash 0-> 5% MeOH in DCM to provide 6MEW160 (55 mg, 98% yield). ^1^H NMR (300 MHz, chloroform-d) δ 7.85 p.p.m. (d, J = 2.5 Hz, 1H), 7.67 (d, J = 8.8 Hz, 1H), 7.47-7.35 (m, 2H), 7.29-7.20 (m, 4H), 7.06 (ddt, J = 6.7, 3.6, 1.8, 1.8 Hz, 2H), 7.00 (s, 1H), 6.90-6.79 (m, 2H), 6.60 (s, 1H), 4.62 (s, 2H), 4.54 (p, J = 6.1, 6.1, 6.1, 6.1 Hz, 1H), 3.44 (tt, J = 7.3, 7.3, 3.0, 3.0 Hz, 5H), 3.24 (s, 2H), 2.49 (ddd, J = 10.3, 6.4, 3.5 Hz, 1H), 2.40 (dt, J = 10.9, 4.8, 4.8 Hz, 1H), 2.31 ? 2.11 (m, 2H), 1.22 (d, J = 6.0 Hz, 4H), 1.14 (d, J = 6.0 Hz, 3H). HRMS (m/z): [M+] cald for C30H32ClN5O4 562.06, found 562.31.

B. Total synthesis of sorafenib and analogs

**Synthesis of Sorafenib and derivatives**

***Chromatography***

Merck pre-coated 0.25 mm silica plates containing a 254 nm fluorescence indicator were used for analytical thin-layer chromatography.

Flash chromatography was performed on 230-400 mesh silica (SiliaFlash P60) from Silicycle.

***Spectroscopy***

NMR spectra were obtained on a Bruker DPX 300 or 400 MHz spectrometer. CI-MS spectra were taken on a Nermag R-10-10 instrument.

***Overall Synthetic Scheme of Sorafenib (SRS13-42) and analog (SRS13-65)***

The starting material, picolinic acid **1**, was first converted to the 4-chloropyridine-2-carbonyl chloride hydrochloride **2** in 90% yield using thionyl chloride and catalytic amount of dimethylformamide (DMF). Amidation of the acid chloride **2** with methylamine and dimethylamine in the presence of triethylamine as HCl acceptor gave the amides **3a** and **3b** in 94% and 90 % yield respectively. In the next reaction step the chloroamides **3a** and **3b** were coupled with 4-aminophenol to give the ethers **4a** and **4b** in 70% and 69% yield respectively. The ether side chain were introduced using potassium *tert*-butoxide in the presence of potassium carbonate. These conditions allowed a chemo-selective ArS_N_2 addition of the phenoxide; therefore the main product was the ether, not the secondary amine. Finally, the urea bond formation were performed at room temperature, in dichloromethane, between the aniline of the ethers **4a** and **4b** and the 4-chloro-3-(fluoromethyl)phenyl isocyanate **5.** The final sorafenib products **4a** and **4b** were isolated in 90% and 92% yield respectively.

***Synthesis of 4-chloro-N-methylpyridine-2-carboxamide (3a and 3b, Scheme 1)***

Anhydrous *N*,*N*-dimethylformamide (0.6 mL) was slowly added to thionyl chloride (18 mL) at a temperature range of 40-50 °C under argon. The solution was stirred in that temperature range for 10 min prior to portion wise addition of picolinic acid (**1**, 6.00 g, 48.74 mmol) over a 5-min period. The solution was heated to 70 °C, and vigorous SO_2_ evolution was observed. A yellow solid precipitated after 17 hours. The mixture was then cooled to room temperature, diluted with toluene (50 mL), and concentrated to 20 mL. The mixture was filtered, washed with toluene (50 mL), and dried under high vacuum for 4 hours to afford the 4-chloropyridine-2-carbonyl chloride **2** as a white solid.

To the acid chloride **2** (500 mg, 2.907 mmol) was added portion wise to methylamine (2.0 M) in tetrahydrofuran (100 mL) at 0 °C under argon. The mixture was stirred at 0 °C for 1 hour then at room temperature for 4 hours, concentrated to near dryness, and dissolved in ethyl acetate (50 mL). The organics were washed with brine, dried over sodium sulfate, and concentrated. The crude reaction mixture was purified by column chromatography (dichloromethane/methanol) to provide the 4-chloro-*N*-methylpyridine-2-carboxamide **3a** (459 mg, 2.701 mmol, 94%) as a yellow, crystalline solid.  ^1^H NMR (DMSO-*d*_6_, 400 MHz): *d* 2.83 (s, 3H); 7.71 (dd, *J* = 2.2, 5.1 Hz, 1H); 8.02 (d, *J* = 2.2, 1H); 8.65 (d, *J* = 5.1 Hz, 1H); 8.83 (br, 1H); MS (APCI+, M+1) 171.68.

To the acid chloride **2** (500 mg, 2.87 mmol) was added portion wise to dimethylamine (2.0 M) in tetrahydrofuran (100 mL) at 0 °C under argon. The mixture was stirred at 0 °C for 1 hour then at room temperature for 4 hours, concentrated to near dryness, and dissolved in ethyl acetate (50 mL). The organics were washed with brine, dried over sodium sulfate, and concentrated. The crude reaction mixture was purified by column chromatography (dichloromethane/methanol) to provide the 4-chloro-*N,N*-dimethylpyridine-2-carboxamide **3b** (459 mg, 2.7 mmol, 94%) as a yellow, crystalline solid.  ^1^H NMR (DMSO-*d*_6_, 400 MHz): *d* 2.83 (s, 6H); 7.71 (dd, *J* = 2.2, 5.1 Hz, 1H); 8.02 (d, *J* = 2.2, 1H); 8.65 (d, *J* = 5.1 Hz, 1H); MS (APCI+, M+1) 184.68.

***Synthesis of 4-(4-aminophenoxy)-N-methylpyridine-2-carboxamide (4a, SRS13-42) and 4-(4-aminophenoxy)-N,N-dimethylpyridine-2-carboxamide (4b, SRS13-65) (Scheme 1)***

A solution of 4-aminophenol (256 mg, 2.35 mmol) in dry *N*,*N*-dimethylformamide (40 mL) was treated with potassium *tert*-butoxide (274 mg, 2.44 mmol), and the reddish-brown mixture was stirred at room temperature for 1 hour. The contents were treated with **3a** and **3b** (400 mg, 2.35 mmol) and potassium carbonate (974 mg, 7.05 mmol) and then heated to 80 °C under argon for 4 h. The mixture was cooled to room temperature and poured into ethyl acetate (100 mL) and brine (400 mL). The combined organics were washed with brine, dried over sodium sulfate, and concentrated. The crude reaction mixture was purified by column chromatography (dichloromethane/methanol) to afford the desired 4-(4-aminophenoxy)-N-methylpyridine-2-carboxamide **4a** (400 mg, 1.65 mmol, 70%) and 4-(4-aminophenoxy)-N,N-dimethylpyridine-2-carboxamide (69%) **4b** after column as a light-brown solid.

**4-(4-aminophenoxy)-N-methylpyridine-2-carboxamide** (70%) **4a:** ^1^H NMR (DMSO-*d*_6_, 400 MHz): *d* 2.79 (d, *J* = 4.8 Hz, 3H); 5.17 (br, 2H); 6.60-6.90 (m 4H); 7.09 (dd, *J* = 2.5, 5.5 Hz, 1H,); 7.38 (d, *J* = 2.5 Hz, 1H); 8.49 (d, *J* = 5.5 Hz, 1H); 8.76 (br, 1H); MS (APCI+, M+1) 244.18.

**4-(4-aminophenoxy)-N,N-dimethylpyridine-2-carboxamide** (69%) **4b:** ^1^H NMR (DMSO-*d*_6_, 400 MHz): *d* 2.79 (s, 6H); 5.17 (br, 2H); 6.60-6.90 (m 4H); 7.09 (dd, *J* = 2.5, 5.5 Hz, 1H,); 7.38 (d, *J* = 2.5 Hz, 1H); 8.49 (d, *J* = 5.5 Hz, 1H; MS (APCI+, M+1) 257.18.

***Synthesis of 4-(4-(3-(4-chloro-3-(trifluoromethyl)phenyl)ureido)phenoxy)-N-methylpyridine-2-carboxamide (6a, SRS13-43) and 4-(4-(3-(4-chloro-3-(trifluoromethyl) phenyl)ureido)phenoxy)-N,N-dimethylpyridine-2-carboxamide (6b, SRS13-65) (Scheme 1)***

**Procedure A**. To the 4-(4-aminophenoxy)-N-methylpyridine-2-carboxamide **4a** (300 mg, 1.234 mmol) in methylene chloride (15 mL) was added a solution of 1-chloro-4-isocyanato-2-(trifluoromethyl)benzene **5** (273 mg, 1.235 mmol) in methylene chloride (15 mL) at 0 °C under argon. The mixture was stirred for 17 hours and then filtered. The solids were washed with methylene chloride and dried under vacuum. The crude reaction mixture was purified by column chromatography (dichloromethane/methanol) to afford **6a** (515 mg, 1.109 mmol, 90%) as a white solid. ^1^H NMR (DMSO*d*_6_, 400 MHz): *d* 2.79 (d, *J* = 4.8 Hz, 3H); 7.19 (m, 3H); 7.41 (d, *J* = 2.5 Hz, 1H); 7.67 (m, 4H); 8.13 (d, *J* = 2.5 Hz, 1H); 8.48 (d, *J* = 5.5 Hz, 1H); 8.78 (br, 1H); 8.97 (s, 1H); 9.24 (s, 1H); MS (APCI+, M+1) 465.19. The NMR spectraum of the sorafenib **6a** is similar to the previously reported data by Bankston et al. (2002). ^[[1]](#endnote-1)^

Similarly the 4-(4-(3-(4-chloro-3-(trifluoromethyl) phenyl)ureido)phenoxy)-N,N-dimethylpyridine-2-carboxamide (62%) **(6b)** was prepared starting from the 4-(4-aminophenoxy)-N,N-dimethylpyridine-2-carboxamide **4b**. ^1^H NMR (DMSO*d*_6_, 400 MHz): *d* 2.79 (s, 6H); 7.19 (m, 3H); 7.41 (d, *J* = 2.5 Hz, 1H); 7.67 (m, 4H); 8.13 (d, *J* = 2.5 Hz, 1H); 8.48 (d, *J* = 5.5 Hz, 1H); 8.97 (s, 1H); 9.24 (s, 1H); MS (APCI+, M+1) 478.29.

**Synthesis of Sorafenib derivatives**

**Synthesis of 4-(4-(3-(2-fluoro-5-(trifluoromethyl)phenyl)ureido)phenoxy)-*N*-methylpyridine-2-carboxamide (SRS13-43, Table 1, entry 1)**

Following the above general **procedure A** with the 4-(4-aminophenoxy)-*N*-methylpyridine-2-carboxamide (30 mg, 0.123 mmol), and 1-fluoro-2-isocyanato-4-(trifluoromethyl)benzene (26.7 mL, 0.185 mmol), the crude reaction mixture was purified by column chromatography (dichloromethane: methanol = 40:1) to provide the desired sorafenib analog **(SRS13-43)** (44.0 mg, 0.098 mmol, 80%). ^1^H NMR (400 MHz, DMSO) δ 9.33 (s, 1H), 8.94 (d, *J* = 2.5 Hz, 1H), 8.79 (d, *J* = 4.8 Hz, 1H), 8.68 – 8.60 (m, 1H), 8.51 (d, *J* = 5.6 Hz, 1H), 7.63 – 7.56 (m, 2H), 7.55 – 7.47 (m, 1H), 7.40 (t, *J* = 6.3 Hz, 2H), 7.23 – 7.17 (m, 2H), 7.15 (dd, *J* = 5.6, 2.6 Hz, 1H), 2.79 (d, *J* = 4.8 Hz, 3H); MS (APCI+, *M+1*) 448.96.

**Synthesis of 4-(4-(3-(2-fluoro-3-(trifluoromethyl)phenyl)ureido)phenoxy)-*N*-methylpyridine-2-carboxamide (SRS13-44, Table 1, entry 2)**

Following the above general **procedure A** with the 4-(4-aminophenoxy)-*N*-methylpyridine-2-carboxamide (30 mg, 0.123 mmol), and 2-fluoro-1-isocyanato-3-(trifluoromethyl)benzene (26.7 μL, 0.185 mmol), the crude reaction mixture was purified by column chromatography (dichloromethane: methanol = 40:1) to provide the desired sorafenib analog **(SRS13-44)** (44.0 mg, 0.098 mmol, 80%).^1^H NMR (400 MHz, DMSO, ppm) δ 9.29 (s, 1H), 8.88 (s, 1H), 8.78 (d, *J* = 4.6 Hz, 1H), 8.49 (dd, *J* = 13.4, 4.3 Hz, 2H), 7.72 – 7.55 (m, 2H), 7.38 (dd, *J* = 12.9, 4.4 Hz, 3H), 7.30 – 7.10 (m, 3H), 5.76 (d, *J* = 1.1 Hz, 1H), 2.90 – 2.75 (m, 3H); MS (APCI+, *M+1*) 448.16.

**Synthesis of 4-(4-(3-(3,5-bis(trifluoromethyl)phenyl)ureido)phenoxy)-*N*-methylpyridine-2-carboxamide (SRS13-45, Table 1, entry 3)**

Following the above general **procedure A** with the 4-(4-aminophenoxy)-*N*-methylpyridine-2-carboxamide (30 mg, 0.123 mmol), and 1-isocyanato-3,5-bis(trifluoromethyl)benzene (26.7 mL, 0.185 mmol), the crude reaction mixture was purified by column chromatography (dichloromethane: methanol = 40:1) to provide the desired sorafenib analog **(SRS13-45)** (52.0 mg, 0.104 mmol, 85%). ^1^H ^1^H NMR (400 MHz, DMSO) δ 9.47 (s, 1H), 9.16 (s, 1H), 8.78 (d, *J* = 4.8 Hz, 1H), 8.51 (d, *J* = 5.6 Hz, 1H), 8.16 (s, 2H), 7.73 – 7.59 (m, 3H), 7.39 (d, *J* = 2.5 Hz, 1H), 7.30 – 7.11 (m, 3H), 2.79 (d, *J* = 4.8 Hz, 3H); MS (APCI+, *M+1*) 498.36.

**Synthesis of 4-(4-(3-(4-bromo-2-(trifluoromethyl)phenyl)ureido)phenoxy)-*N*-methylpyridine-2-carboxamide (SRS13-46, Table 1, entry 4)**

Following the above general **procedure A** with the 4-(4-aminophenoxy)-*N*-methylpyridine-2-carboxamide (30 mg, 0.123 mmol), and 4-bromo-1-isocyanato-2-(trifluoromethyl)benzene (28.8 mL, 0.185 mmol), the crude reaction mixture was purified by column chromatography (dichloromethane: methanol = 40:1) to provide the desired sorafenib analog **(SRS13-46)** (56.0 mg, 0.110 mmol, 89%). ^1^H NMR (400 MHz, DMSO, ppm) δ 9.58 (s, 1H), 8.79 (d, *J* = 4.8 Hz, 1H), 8.51 (d, *J* = 5.6 Hz, 1H), 8.20 (s, 1H), 7.97 (d, *J* = 9.4 Hz, 1H), 7.93 – 7.78 (m, 2H), 7.59 (d, *J* = 8.9 Hz, 2H), 7.39 (d, *J* = 2.3 Hz, 1H), 7.28 – 7.11 (m, 3H), 2.79 (d, *J* = 4.8 Hz, 3H); MS (APCI+, *M+1*) 509.36.

**Synthesis of 4-(4-(3-(3-chlorophenyl)ureido)phenoxy)-*N*-methylpyridine-2-carboxamide (SRS13-47, Table 1, entry 5)**

Following the above general **procedure A** with the 4-(4-aminophenoxy)-*N*-methylpyridine-2-carboxamide (30 mg, 0.123 mmol), and 1-chloro-3-isocyanatobenzene (28.8 mL, 0.185 mmol), the crude reaction mixture was purified by column chromatography (dichloromethane: methanol = 40:1) to provide the desired sorafenib analog **(SRS13-47)** (33.7 mg, 0.110 mmol, 69%). ^1^H NMR (400 MHz, DMSO) δ 8.93 (d, *J* = 11.9 Hz, 2H), 8.79 (d, *J* = 4.8 Hz, 1H), 8.51 (d, *J* = 5.6 Hz, 1H), 7.73 (d, *J* = 1.8 Hz, 1H), 7.69 – 7.55 (m, 2H), 7.38 (d, *J* = 2.6 Hz, 1H), 7.37 – 7.27 (m, 2H), 7.16 (ddd, *J* = 8.9, 6.3, 2.4 Hz, 3H), 7.03 (dt, *J* = 6.9, 2.1 Hz, 1H), 2.79 (d, *J* = 4.8 Hz, 3H); MS (APCI+, *M+1*) 396.16.

**Synthesis of 4-(4-(3-(4-chlorophenyl)ureido)phenoxy)-*N*-methylpyridine-2-carboxamide (SRS13-48, Table 1, entry 6)**

Following the above general **procedure A** with the 4-(4-aminophenoxy)-*N*-methylpyridine-2-carboxamide (30 mg, 0.123 mmol), and 1-chloro-4-isocyanatobenzene (22.7 mL, 0.185 mmol), the crude reaction mixture was purified by column chromatography (dichloromethane: methanol = 40:1) to provide the desired sorafenib analog **(SRS13-48)** (36.5 mg, 0.110 mmol, 75%). ^1^H NMR (400 MHz, DMSO) δ 8.88 (s, 2H), 8.79 (d, *J* = 4.8 Hz, 1H), 8.51 (d, *J* = 5.6 Hz, 1H), 7.59 (s, 1H), 7.57 – 7.47 (m, 3H), 7.46 – 7.29 (m, 3H), 7.28 – 7.12 (m, 3H), 2.79 (d, *J* = 4.8 Hz, 3H); MS (APCI+, *M+1*) 396.16.

**Synthesis of 4-(4-(3-(4-chloro-2-(trifluoromethyl)phenyl)ureido)phenoxy)-*N*-methylpyridine-2-carboxamide (SRS13-49, Table 1, entry 7)**

Following the above general **procedure A** with the 4-(4-aminophenoxy)-*N*-methylpyridine-2-carboxamide (30 mg, 0.123 mmol), and 4-chloro-1-isocyanato-2-(trifluoromethyl)benzene (22.7 mL, 0.185 mmol), the crude reaction mixture was purified by column chromatography (dichloromethane: methanol = 40:1) to provide the desired sorafenib analog **(SRS13-49)** (46.0 mg, 0.099 mmol, 80%). ^1^H ^1^H NMR (400 MHz, DMSO) δ 9.59 (s, 1H), 8.78 (d, *J* = 4.6 Hz, 1H), 8.50 (d, *J* = 5.6 Hz, 1H), 8.21 (s, 1H), 8.02 (d, *J* = 8.5 Hz, 1H), 7.72 (d, *J* = 9.1 Hz, 2H), 7.60 (d, *J* = 8.1 Hz, 2H), 7.40 (s, 1H), 7.30 – 7.10 (m, 3H), 2.79 (d, *J* = 4.2 Hz, 3H); MS (APCI+, *M+1*) 464.16.

**Synthesis of *N*-methyl-4-(4-(3-(4-(trifluoromethyl)phenyl)ureido)phenoxy)pyridine-2-carboxamide (SRS13-52, Table 1, entry 8)**

Following the above general **procedure A** with the 4-(4-aminophenoxy)-*N*-methylpyridine-2-carboxamide (30 mg, 0.123 mmol), and 1-isocyanato-4-(trifluoromethyl)benzene (26.4 mL, 0.185 mmol), the crude reaction mixture was purified by column chromatography (dichloromethane: methanol = 40:1) to provide the desired sorafenib analog **(SRS13-52)** (44.0 mg, 0.102 mmol, 83%). ^1^H NMR (400 MHz, DMSO) δ 9.20 (s, 1H), 9.00 (s, 1H), 8.79 (d, *J* = 4.7 Hz, 1H), 8.51 (d, *J* = 5.6 Hz, 1H), 7.65 (dt, *J* = 14.9, 8.8 Hz, 6H), 7.40 (d, *J* = 2.4 Hz, 1H), 7.29 – 7.12 (m, 3H), 2.79 (d, *J* = 4.7 Hz, 3H); MS (APCI+, *M+1*) 430.19.

**Synthesis of 4-(4-(3-(2-chlorophenyl)ureido)phenoxy)-*N*-methylpyridine-2-carboxamide (SRS13-53, Table 1, entry 9)**

Following the above general **procedure A** with the 4-(4-aminophenoxy)-*N*-methylpyridine-2-carboxamide (30 mg, 0.123 mmol), and 1-chloro-2-isocyanatobenzene (22.3 mL, 0.185 mmol), the crude reaction mixture was purified by column chromatography (dichloromethane: methanol = 40:1) to provide the desired sorafenib analog **(SRS13-53)** (46.4 mg, 0.117 mmol, 95%). ^1^H NMR (400 MHz, DMSO) δ 9.94 (s, 1H), 8.59 – 8.38 (m, 2H), 8.26 – 7.95 (m, 2H), 7.63 (d, *J* = 8.9 Hz, 2H), 7.61 – 7.42 (m, 2H), 7.42 – 7.25 (m, 1H), 7.19 (dd, *J* = 5.9, 2.5 Hz, 3H), 7.16 – 6.98 (m, 1H), 2.80 (d, *J* = 4.7 Hz, 3H); MS (APCI+, *M+1*) 396.18.

**Synthesis of *N*-methyl-4-(4-(3-(3-(trifluoromethyl)phenyl)ureido)phenoxy)pyridine-2-carboxamide (SRS13-54, Table 1, entry 10)**

Following the above general procedure A with the 4-(4-aminophenoxy)-N-methylpyridine-2-carboxamide (30 mg, 0.123 mmol), and 1-chloro-2-isocyanatobenzene (22.3 mL, 0.185 mmol), the crude reaction mixture was purified by column chromatography (dichloromethane: methanol = 40:1) to provide the desired sorafenib analog (SRS13-54) (45.0 mg, 0.104 mmol, 85%). 1H NMR (400 MHz, DMSO) δ 9.16 (s, 1H), 9.01 (s, 1H), 8.80 (d, J = 4.8 Hz, 1H), 8.51 (d, J = 5.6 Hz, 1H), 8.04 (s, 1H), 7.61 (d, J = 8.9 Hz, 3H), 7.52 (t, J = 7.9 Hz, 1H), 7.41 (d, J = 2.5 Hz, 1H), 7.32 (d, J = 7.6 Hz, 1H), 7.27 – 7.12 (m, 3H), 5.76 (s, 1H), 2.80 (d, J = 4.8 Hz, 3H); MS (APCI+, M+1) 430.19.

**Synthesis of 4-(4-(3-(3-cyanophenyl)ureido)phenoxy)-*N*-methylpyridine-2-carboxamide (SRS13-57, Table 1, entry 11)**

Following the above general **procedure A** with the 4-(4-aminophenoxy)-*N*-methylpyridine-2-carboxamide (30 mg, 0.123 mmol), and 3-isocyanatobenzonitrile (26.6 mg, 0.185 mmol), the crude reaction mixture was purified by column chromatography (dichloromethane: methanol = 40:1) to provide the desired sorafenib analog **(SRS13-57)** (41.0 mg, 0.106 mmol, 86%). ^1^H NMR (400 MHz, DMSO) δ 9.14 (s, 1H), 9.07 (d, *J* = 32.6 Hz, 2H), 8.79 (d, *J* = 4.8 Hz, 1H), 8.51 (d, *J* = 5.6 Hz, 1H), 7.99 (d, *J* = 1.4 Hz, 1H), 7.70 (dd, *J* = 8.2, 1.0 Hz, 1H), 7.61 (s, 1H), 7.60 – 7.35 (m, 4H), 7.30 – 7.12 (m, 3H), 2.79 (d, *J* = 4.8 Hz, 3H); MS (APCI+, *M+1*) 387.39.

**Synthesis of *N*-methyl-4-(4-(3-phenylureido)phenoxy)pyridine-2-carboxamide (SRS13-59, Table 1, entry 12)**

Following the above general **procedure A** with the 4-(4-aminophenoxy)-*N*-methylpyridine-2-carboxamide (30 mg, 0.123 mmol), and isocyanatobenzene (25 μL, 0.185 mmol), the crude reaction mixture was purified by column chromatography (dichloromethane: methanol = 40:1) to provide the desired sorafenib analog **(SRS13-59)** (39.0 mg, 0.107 mmol, 87%). ^1^H NMR (400 MHz, DMSO): *d* 2.79 (d, *J* = 4.8 Hz, 3H); 7.19 (m, 5H); 7.41 (d, *J* = 2.5 Hz, 1H); 7.67 (m, 4H); 8.13 (d, *J* = 2.5 Hz, 1H); 8.48 (d, *J* = 5.5 Hz, 1H); 8.78 (br, 1H); 8.97 (s, 1H); 9.24 (s, 1H); MS (APCI+, M+1) 463.09; MS (APCI+, *M+1*) 362.19.

**Synthesis of *N*-methyl-4-(4-(3-(2-(trifluoromethyl)phenyl)ureido)phenoxy)pyridine-2-carboxamide (SRS13-67, Table 1, entry 13)**

Following the above general **procedure A** with the 4-(4-aminophenoxy)-*N*-methylpyridine-2-carboxamide (30 mg, 0.123 mmol), and 1-isocyanato-2-(trifluoromethyl)benzene (18 mL, 0.185 mmol), the crude reaction mixture was purified by column chromatography (dichloromethane: methanol = 40:1) to provide the desired sorafenib analog **(SRS13-67)** (51.0 mg, 0.119 mmol, 97%). ^1^H NMR (400 MHz, DMSO) δ 9.52 (s, 1H), 8.76 (d, *J* = 4.6 Hz, 1H), 8.50 (t, *J* = 5.1 Hz, 1H), 8.12 (s, 1H), 7.96 (d, *J* = 8.1 Hz, 1H), 7.72 – 7.63 (m, 2H), 7.59 (t, *J* = 6.8 Hz, 2H), 7.42 – 7.36 (m, 1H), 7.30 (t, *J* = 7.2 Hz, 1H), 7.21 – 7.13 (m, 3H), 2.79 (t, *J* = 4.7 Hz, 3H); MS (APCI+, *M+1*) 431.19.

**Synthesis of 4-(4-(3-(4-chloro-3-(trifluoromethyl)phenyl)thioureido)phenoxy)-*N*-methylpyridine-2-carboxamide (SRS13-60, Table 1, entry 14)**

Following the above general **procedure A** with the 4-(4-aminophenoxy)-*N*-methylpyridine-2-carboxamide (30 mg, 0.123 mmol), and 1-chloro-4-isothiocyanato-2-(trifluoromethyl)benzene (30 mL, 0.185 mmol), the crude reaction mixture was purified by column chromatography (dichloromethane: methanol = 40:1) to provide the desired sorafenib analog **(SRS13-60)** (52.0 mg, 0.108 mmol, 88%). ^1^H NMR (400 MHz, DMSO) δ 10.16 (s, 2H), 8.78 (d, *J* = 4.8 Hz, 1H), 8.54 (d, *J* = 5.6 Hz, 1H), 8.10 (d, *J* = 2.4 Hz, 1H), 7.83 (dd, *J* = 8.7, 2.4 Hz, 1H), 7.69 (d, *J* = 8.7 Hz, 1H), 7.59 (d, *J* = 8.8 Hz, 2H), 7.44 (d, *J* = 2.5 Hz, 1H), 7.27 – 7.13 (m, 3H), 2.80 (d, *J* = 4.8 Hz, 3H); MS (APCI+, *M+1*) 480.79.

**Synthesis of 1-(4-chloro-3-(trifluoromethyl)phenyl)-3-(4-phenoxyphenyl)urea (SRS13-97, Table 2, entry 1)**

Following the above general **procedure A** with the 4-phenoxyaniline (30 mg, 0.162 mmol), and 1-chloro-4-isocyanato-2-(trifluoromethyl)benzene (53 mg, 0.243 mmol), the crude reaction mixture was purified by column chromatography (dichloromethane: methanol = 100:1) to provide the desired sorafenib analog **(SRS13-97)** (52.0 mg, 0.128 mmol, 80%). ^1^H NMR (400 MHz, DMSO) δ 9.15 (s, 2H), 8.85 (s, 2H), 8.12 (s, 2H), 7.62 (dd, *J* = 25.1, 8.8 Hz, 4H), 7.50 (d, *J* = 7.5 Hz, 4H), 7.36 (t, *J* = 7.2 Hz, 4H), 7.09 (t, *J* = 7.3 Hz, 2H), 6.98 (t, *J* = 9.5 Hz, 8H); MS (APCI+, *M+1*) 406.49.

**Synthesis of 1-(4-chloro-3-(trifluoromethyl)phenyl)-3-(4-phenoxyphenyl)thiourea (SRS13-98, Table 2, entry 2)**

Following the above general **procedure A** with the 4-phenoxyaniline (30 mg, 0.162 mmol), and 1-chloro-4-isothiocyanato-2-(trifluoromethyl)benzene (53 mg, 0.243 mmol), the crude reaction mixture was purified by column chromatography (dichloromethane: methanol = 100:1) to provide the desired sorafenib analog **(SRS13-98)** (60.0 mg, 0.142 mmol, 88%). ^1^H NMR (400 MHz, DMSO) δ 10.05 (s, 2H), 8.09 (d, *J* = 2.3 Hz, 1H), 7.81 (dd, *J* = 8.7, 2.3 Hz, 1H), 7.66 (d, *J* = 8.7 Hz, 1H), 7.52 – 7.29 (m, 4H), 7.15 (t, *J* = 7.4 Hz, 1H), 7.02 (dd, *J* = 8.7, 2.3 Hz, 4H); MS (APCI+, *M+1*) 423.1.

**Synthesis of 1-(4-chloro-3-(trifluoromethyl)phenyl)-3-(4-(4 cyanophenoxy)phenyl)-urea (SRS14-22, Table 2, entry 3)**

Following the above general **procedure A** with the 4-(4-aminophenoxy)benzonitrile (25 mg, 0.119 mmol), and 1-chloro-4-isocyanato-2-(trifluoromethyl)benzene (39.5 mg, 0.178 mmol), the crude reaction mixture was purified by column chromatography (dichloromethane: methanol = 50:1) to provide the desired sorafenib analog **(SRS14-22)** (46.0 mg, 0.106 mmol, 90%). ^1^H NMR (400 MHz, DMSO) δ 9.13 (s, 1H), 8.81 (s, 1H), 8.11 (d, *J* = 2.3 Hz, 1H), 7.63 (dt, *J* = 15.0, 5.6 Hz, 2H), 7.49 – 7.42 (m, 2H), 7.17 (d, *J* = 8.3 Hz, 2H), 6.98 – 6.84 (m, 4H); MS (APCI+, *M+1*) 431.19.

**Synthesis of 1-(4-chloro-3-(trifluoromethyl)phenyl)-3-(4-(4-cyanophenoxy)phenyl)-thiourea (SRS14-26, Table 2, entry 4)**

Following the above general **procedure A** with the 4-(4-aminophenoxy)benzonitrile (25 mg, 0.119 mmol), and 1-chloro-4-isothiocyanato-2-(trifluoromethyl)benzene (42.0 mg, 0.178 mmol), the crude reaction mixture was purified by column chromatography (dichloromethane: methanol = 50:1) to provide the desired sorafenib analog **(SRS14-26)** (42.0 mg, 0.094 mmol, 79%). ^1^H NMR (400 MHz, DMSO) δ 10.02 (d, *J* = 15.8 Hz, 2H), 8.08 (s, 1H), 7.90 – 7.75 (m, 1H), 7.65 (d, *J* = 8.7 Hz, 1H), 7.41 (d, *J* = 8.8 Hz, 2H), 7.20 (d, *J* = 8.3 Hz, 2H), 6.94 (dd, *J* = 14.7, 8.6 Hz, 4H); MS (APCI+, *M+1*) 447.19.

**Synthesis of 1-(4-chloro-3-(trifluoromethyl)phenyl)-3-(4-(3-cyanophenoxy)phenyl)-urea (SRS14-23, Table 2, entry 5)**

Following the above general **procedure A** with the 3-(4-aminophenoxy)benzonitrile (25 mg, 0.119 mmol), and 1-chloro-4-isocyanato-2-(trifluoromethyl)benzene (39.5 mg, 0.178 mmol), the crude reaction mixture was purified by column chromatography (dichloromethane: methanol = 50:1) to provide the desired sorafenib analog **(SRS14-23)** (46.0 mg, 0.106 mmol, 90%).^1^H NMR (400 MHz, DMSO) δ 9.15 (s, 1H), 8.84 (s, 1H), 8.11 (s, 1H), 7.63 (dt, *J* = 14.9, 5.6 Hz, 2H), 7.47 (d, *J* = 8.9 Hz, 2H), 7.24 (t, *J* = 7.8 Hz, 1H), 6.95 (dd, *J* = 25.4, 8.2 Hz, 3H), 6.87 – 6.70 (m, 2H); MS (APCI+, *M+1*) 431.19.

**Synthesis of 1-(4-chloro-3-(trifluoromethyl)phenyl)-3-(4-(3-cyanophenoxy)phenyl)-thiourea (SRS14-27, Table 2, entry 6)**

Following the above general **procedure A** with the 3-(4-aminophenoxy)benzonitrile (25 mg, 0.119 mmol), and 1-chloro-4-isothiocyanato-2-(trifluoromethyl)benzene (42.0 mg, 0.178 mmol), the crude reaction mixture was purified by column chromatography (dichloromethane: methanol = 50:1) to provide the desired sorafenib analog **(SRS14-27)** (42.0 mg, 0.094 mmol, 79%). ^1^H NMR (400 MHz, DMSO) δ 10.03 (s, 2H), 8.08 (s, 1H), 7.81 (d, *J* = 8.6 Hz, 1H), 7.66 (d, *J* = 8.7 Hz, 1H), 7.43 (d, *J* = 8.7 Hz, 2H), 7.27 (t, *J* = 7.8 Hz, 1H), 6.98 (dd, *J* = 12.8, 8.3 Hz, 3H), 6.90 – 6.73 (m, 2H); MS (APCI+, *M+1*) 447.19.

**Synthesis of 1-(4-chloro-3-(trifluoromethyl)phenyl)-3-(4-(3-morpholinophenoxy)phenyl) urea (SRS14-30, Table 2, entry 7)**

Following the above general procedure A with the 4-(3-morpholinophenoxy)aniline (37 mg, 0.137 mmol), and 1-chloro-4-isocyanato-2-(trifluoromethyl)benzene (45.0 mg, 0.205 mmol), the crude reaction mixture was purified by column chromatography (dichloromethane: methanol = 50:1) to provide the desired sorafenib analog (**SRS14-30**) (45.0 mg, 0.094 mmol, 79%). ^1^H NMR (400 MHz, DMSO) δ 9.13 (s, 1H), 8.82 (s, 1H), 8.11 (d, *J* = 2.3 Hz, 1H), 7.62 (dd, *J* = 15.2, 5.5 Hz, 2H), 7.47 (d, *J* = 9.0 Hz, 2H), 7.18 (t, *J* = 8.2 Hz, 1H), 6.97 (d, *J* = 8.9 Hz, 2H), 6.68 (dd, *J* = 8.3, 2.2 Hz, 1H), 6.57 (t, *J* = 2.2 Hz, 1H), 6.34 (dd, *J* = 7.9, 1.9 Hz, 1H), 3.76 – 3.66 (m, 4H), 3.13 – 3.02 (m, 4H); MS (APCI+, *M+1*) 491.19.

**Synthesis of 1-(4-chloro-3-(trifluoromethyl)phenyl)-3-(4-(3 morpholinophenoxy)-phenyl) thiourea (SRS14-31, Table 2, entry 8)**

Following the above general **procedure A** with the 4-(3-morpholinophenoxy)aniline (30 mg, 0.111 mmol), and 1-chloro-4-isothiocyanato-2-(trifluoromethyl)benzene (39.0 mg, 0.166 mmol), the crude reaction mixture was purified by column chromatography (dichloromethane: methanol = 40:1) to provide the desired sorafenib analog **(SRS14-31)** (46.0 mg, 0.090 mmol, 82%). ^1^H NMR (400 MHz, DMSO) δ 10.05 (s, 1H), 9.99 (s, 1H), 8.08 (d, *J* = 2.4 Hz, 1H), 7.81 (dd, *J* = 8.7, 2.5 Hz, 1H), 7.66 (d, *J* = 8.7 Hz, 1H), 7.42 (d, *J* = 8.8 Hz, 2H), 7.22 (t, *J* = 8.2 Hz, 1H), 7.02 – 6.95 (m, 2H), 6.74 (dd, *J* = 8.3, 2.2 Hz, 1H), 6.62 (t, *J* = 2.2 Hz, 1H), 6.39 (dd, *J* = 8.0, 2.0 Hz, 1H), 3.76 – 3.66 (m, 4H), 3.15 – 3.05 (m, 4H); MS (APCI+, *M+1*) 507.19.

**Synthesis of 1-(3,5-bis(trifluoromethyl)phenyl)-3-(4-(3-morpholinophenoxy)-phenyl)urea (SRS14-29, Table 2, entry 9)**

Following the above general procedure A with the 4-(3-morpholinophenoxy)aniline (30 mg, 0.111 mmol), and 1-isocyanato-3,5-bis(trifluoromethyl)benzene (42.0 mg, 0.166 mmol), the crude reaction mixture was purified by column chromatography (dichloromethane: methanol = 40:1) to provide the desired sorafenib analog (**SRS14-29**) (52.0 mg, 0.090 mmol, 89%). ^1^H NMR (400 MHz, DMSO) δ 9.37 (s, 1H), 8.97 (s, 1H), 8.14 (s, 2H), 7.62 (s, 1H), 7.48 (d, *J* = 8.8 Hz, 2H), 7.19 (t, *J* = 8.2 Hz, 1H), 6.98 (d, *J* = 8.8 Hz, 2H), 6.69 (d, *J* = 8.3 Hz, 1H), 6.57 (s, 1H), 6.35 (d, *J* = 8.0 Hz, 1H), 3.77 – 3.65 (m, 4H), 3.14 – 3.02 (m, 4H); MS (APCI+, *M+1*) 525.29.

**Synthesis of 1-(3,5-bis(trifluoromethyl)phenyl)-3-(4-(4-cyanophenoxy)phenyl)urea (SRS14-24, Table 2, entry 10)**

Following the above general **procedure A** with the 4-(4-aminophenoxy)benzonitrile (25 mg, 0.119 mmol), and 1-isocyanato-3,5-bis(trifluoromethyl)benzene (45.5 mg, 0.178 mmol), the crude reaction mixture was purified by column chromatography (dichloromethane: methanol = 50:1) to provide the desired sorafenib analog **(SRS14-24)** (49.0 mg, 0.105 mmol, 88%). ^1^H NMR (400 MHz, DMSO) δ 9.37 (s, 1H), 8.96 (s, 1H), 8.14 (s, 2H), 7.62 (s, 1H), 7.48 (d, *J* = 7.0 Hz, 2H), 7.17 (d, *J* = 6.9 Hz, 2H), 7.00 – 6.76 (m, 4H); MS (APCI+, *M+1*) 466.05.

**Synthesis of 1-(3,5-bis(trifluoromethyl)phenyl)-3-(4-(3-cyanophenoxy)phenyl)urea (SRS14-25, Table 2, entry 11)**

Following the above general **procedure A** with the 3-(4-aminophenoxy)benzonitrile (25 mg, 0.119 mmol), and 1-isocyanato-3,5-bis(trifluoromethyl)benzene (30.0 mg, 0.178 mmol), the crude reaction mixture was purified by column chromatography (dichloromethane: methanol = 50:1) to provide the desired sorafenib analog **(SRS14-25)** (49.0 mg, 0.099 mmol, 89%). ^1^H NMR (400 MHz, DMSO) δ 9.42 (s, 1H), 9.02 (s, 1H), 8.15 (s, 2H), 7.64 (s, 1H), 7.50 (d, *J* = 8.9 Hz, 2H), 7.25 (t, *J* = 7.7 Hz, 1H), 6.99 (d, *J* = 8.8 Hz, 2H), 6.92 (d, *J* = 7.2 Hz, 1H), 6.82 – 6.70 (m, 2H); MS (APCI+, *M+1*) 465.29.

**Synthesis of 1-(4-benzylphenyl)-3-(3,5-bis(trifluoromethyl)phenyl)urea (SRS14-32, Table 2, entry 12)**

Following the above general **procedure A** with the 4-benzylaniline (30 mg, 0.163 mmol), and 1-isocyanato-3,5-bis(trifluoromethyl)benzene (63.0 mg, 0.245 mmol), the crude reaction mixture was purified by column chromatography (dichloromethane: methanol = 100:1) to provide the desired sorafenib analog **(SRS14-32)** (64.0 mg, 0.146 mmol, 89%). ^1^H NMR (400 MHz, DMSO) δ 9.35 (s, 1H), 8.91 (s, 1H), 8.12 (s, 2H), 7.62 (s, 1H), 7.43 – 7.11 (m, 9H), 3.89 (s, 2H); MS (APCI+, *M+1*) 438.19.

**Synthesis of 1-(4-benzylphenyl)-3-(3,5-bis(trifluoromethyl)phenyl)thiourea (SRS14-33, Table 2, entry 13)**

Following the above general procedure A with the 4-benzylaniline (30 mg, 0.163 mmol), and 1-chloro-4-isothiocyanato-2-(trifluoromethyl)benzene (58.0 mg, 0.245 mmol), the crude reaction mixture was purified by column chromatography (dichloromethane: methanol = 100:1) to provide the desired sorafenib analog (**SRS14-33**) (60.0 mg, 0.142 mmol, 87%). 1H NMR (400 MHz, DMSO) δ 10.06 (s, 1H), 10.01 (s, 1H), 8.09 (d, J = 2.5 Hz, 1H), 7.80 (dd, J = 8.7, 2.4 Hz, 1H), 7.65 (d, J = 8.7 Hz, 1H), 7.41 – 7.15 (m, 9H); MS (APCI+, M+1) 420.29.

**Synthesis of 1-(4-benzylphenyl)-3-(4-chloro-3-(trifluoromethyl)phenyl)urea (SRS14-34, Table 2, entry 14)**

Following the above general **procedure A** with the 4-benzylaniline (30 mg, 0.163 mmol), and 1-chloro-4-isocyanato-2-(trifluoromethyl)benzene (58.0 mg, 0.245 mmol), the crude reaction mixture was purified by column chromatography (dichloromethane: methanol = 100:1) to provide the desired sorafenib analog **(SRS14-34)** (60.0 mg, 0.150 mmol, 92%). ^1^H NMR (400 MHz, DMSO) δ 9.11 (s, 1H), 8.76 (s, 1H), 8.11 (s, 1H), 7.61 (dd, *J* = 22.7, 8.8 Hz, 2H), 7.39 (d, *J* = 8.1 Hz, 2H), 7.22 (ddd, *J* = 33.1, 17.7, 7.7 Hz, 7H), 3.88 (s, 2H); MS (APCI+, *M+1*) 405.19.

**Synthesis of 1-(3,5-bis(trifluoromethyl)phenyl)-3-(4-morpholinophenyl)urea (SRS14-04, Table 3, entry 1)**

Following the above general **procedure A** with the 4-morpholinoaniline (50 mg, 0.280 mmol), and 1-isocyanato-3,5-bis(trifluoromethyl)benzene (107.4 mg, 0.421 mmol), the crude reaction mixture was purified by column chromatography (dichloromethane: methanol = 100:1) to provide the desired sorafenib analog **(SRS14-04)** (103.0 mg, 0.237 mmol, 85%). ^1^H NMR (400 MHz, DMSO) δ 9.29 (s, 1H), 8.70 (s, 1H), 8.13 (s, 2H), 7.58 (s, 1H), 7.35 (d, *J* = 9.0 Hz, 2H), 6.90 (d, *J* = 9.0 Hz, 2H), 3.38 (s, 4H), 3.03 (d, *J* = 4.6 Hz, 4H); MS (APCI+, *M+1*) 433.28.

**Synthesis of 1-(4-chloro-3-(trifluoromethyl)phenyl)-3-(4-morpholinophenyl)urea (SRS14-05, Table 3, entry 2)**

Following the above general **procedure A** with the 4-morpholinoaniline (50 mg, 0.280 mmol), and 1-chloro-4-isocyanato-2-(trifluoromethyl)benzene (93.0 mg, 0.421 mmol), the crude reaction mixture was purified by column chromatography (dichloromethane: methanol = 100:1) to provide the desired sorafenib analog **(SRS14-05)** (94.0 mg, 0.235 mmol, 84%). ^1^H NMR (400 MHz, DMSO) δ 9.05 (s, 1H), 8.57 (s, 1H), 8.11 (s, 1H), 7.60 (dd, *J* = 22.2, 8.8 Hz, 2H), 7.33 (d, *J* = 8.9 Hz, 2H), 6.89 (d, *J* = 8.9 Hz, 2H), 3.38 (s, 4H), 3.10 – 2.98 (m, 4H); MS (APCI+, *M+1*) 399.19.

**Synthesis of 1-(3,5-bis(trifluoromethyl)phenyl)-3-(4-(pyridin-4-ylmethyl)phenyl)-urea (SRS14-10, Table 3, entry 3)**

Following the above general **procedure A** with the 4-(pyridin-4-ylmethyl)aniline (30 mg, 0.163 mmol), and 1-isocyanato-3,5-bis(trifluoromethyl)benzene (62.0 mg, 0.244 mmol), the crude reaction mixture was purified by column chromatography (dichloromethane: methanol = 40:1) to provide the desired sorafenib analog **(SRS14-10)** (65.0 mg, 0.148 mmol, 90%). ^1^H NMR (400 MHz, DMSO) δ 9.37 (s, 1H), 8.94 (s, 1H), 8.46 (d, *J* = 5.1 Hz, 2H), 8.13 (s, 2H), 7.60 (s, 1H), 7.44 (d, *J* = 8.2 Hz, 2H), 7.21 (dd, *J* = 16.0, 6.7 Hz, 4H), 3.91 (s, 2H); MS (APCI+, *M+1*) 439.19.

**Synthesis of 1-(4-chloro-3-(trifluoromethyl)phenyl)-3-(4-(pyridin-4-ylmethyl)-phenyl)urea (SRS14-11, Table 3, entry 4)**

Following the above general **procedure A** with the 4-(pyridin-4-ylmethyl)aniline (30 mg, 0.163 mmol), and 1-chloro-4-isocyanato-2-(trifluoromethyl)benzene (54.0 mg, 0.244 mmol), the crude reaction mixture was purified by column chromatography (dichloromethane: methanol = 40:1) to provide the desired sorafenib analog **(SRS14-11)** (61.0 mg, 0.150 mmol, 92%). ^1^H NMR (400 MHz, DMSO) δ 9.13 (s, 1H), 8.80 (s, 1H), 8.50 – 8.36 (m, 2H), 8.10 (s, 1H), 7.61 (s, 2H), 7.40 (d, *J* = 8.5 Hz, 2H), 7.21 (dd, *J* = 23.1, 7.1 Hz, 4H), 3.92 (s, 2H); MS (APCI+, *M+1*) 406 .01.

**Synthesis of 1-(3,5-bis(trifluoromethyl)phenyl)-3-(6-morpholinopyridin-3-yl)urea (SRS14-12, Table 3, entry 5)**

Following the above general **procedure A** with the 6-morpholinopyridin-3-amine (25 mg, 0.139 mmol), and 1-isocyanato-3,5-bis(trifluoromethyl)benzene (53.0 mg, 0.209 mmol), the crude reaction mixture was purified by column chromatography (dichloromethane: methanol = 20:1) to provide the desired sorafenib analog **(SRS14-12)** (57.0 mg, 0.150 mmol, 95%). ^1^H NMR (400 MHz, DMSO) δ 9.40 (s, 1H), 8.74 (s, 1H), 8.19 (d, *J* = 2.5 Hz, 1H), 8.14 (s, 2H), 7.73 (dd, *J* = 9.0, 2.7 Hz, 1H), 7.62 (s, 1H), 6.84 (d, *J* = 9.0 Hz, 1H), 3.77 – 3.64 (m, 4H), 3.38 (d, *J* = 5.0 Hz, 4H); MS (APCI+, *M+1*) 435.2.

**Synthesis of 1-(4-chloro-3-(trifluoromethyl)phenyl)-3-(6-morpholinopyridin-3-yl)urea (SRS14-13, Table 3, entry 6)**

Following the above general **procedure A** with the 6-morpholinopyridin-3-amine (25 mg, 0.139 mmol), and 1-chloro-4-isocyanato-2-(trifluoromethyl)benzene (46.0 mg, 0.209 mmol), the crude reaction mixture was purified by column chromatography (dichloromethane: methanol = 20:1) to provide the desired sorafenib analog **(SRS14-13)** (50.0 mg, 0.150 mmol, 90%). ^1^H NMR (400 MHz, DMSO) δ 9.16 (s, 1H), 8.59 (s, 1H), 8.18 (d, *J* = 2.6 Hz, 1H), 8.09 (d, *J* = 2.4 Hz, 1H), 7.71 (dd, *J* = 9.0, 2.7 Hz, 1H), 7.62 (dd, *J* = 16.4, 5.6 Hz, 2H), 6.83 (d, *J* = 9.1 Hz, 1H), 3.76 – 3.64 (m, 4H), 3.37 (d, *J* = 5.0 Hz, 4H); MS (APCI+, *M+1*) 435.11.

**Synthesis of 1-(4-chloro-3-(trifluoromethyl)phenyl)-3-(4-(morpholinomethyl)-phenyl)urea (SRS14-66, Table 3, entry 7)**

Following the above general **procedure A** with the 4-(morpholinomethyl)aniline (25 mg, 0.130 mmol), and 1-chloro-4-isocyanato-2-(trifluoromethyl)benzene (46.0 mg, 0.195 mmol), the crude reaction mixture was purified by column chromatography (dichloromethane: methanol = 20:1) to provide the desired sorafenib analog **(SRS14-66)** (47.0 mg, 0.113 mmol, 88%). ^1^H NMR (400 MHz, CDCl_3_) δ 9.65 (s, 1H), 9.33 (s, 1H), 8.63 (s, 1H), 8.21 – 8.10 (m, 2H), 7.93 (d, *J* = 8.3 Hz, 2H), 7.74 (d, *J* = 8.2 Hz, 2H), 4.09 (d, *J* = 3.8 Hz, 4H), 3.91 (s, 2H), 2.85 (s, 4H); MS (APCI+, *M+1*) 413 .29.

**Synthesis of 1-(3,5-bis(trifluoromethyl)phenyl)-3-(4-(morpholinomethyl)-phenyl)urea (SRS14-67, Table 3, entry 8)**

Following the above general **procedure A** with the 4-(morpholinomethyl)aniline (25 mg, 0.130 mmol), and 1-isocyanato-3,5-bis(trifluoromethyl)benzene (49.7 mg, 0.195 mmol), the crude reaction mixture was purified by column chromatography (dichloromethane: methanol = 20:1) to provide the desired sorafenib analog **(SRS14-67)** (51.0 mg, 0.113 mmol, 87%). ^1^H ^1^H NMR (400 MHz, CDCl_3_) δ 9.93 (s, 1H), 9.51 (s, 1H), 8.66 (s, 2H), 8.17 (s, 1H), 7.97 (d, *J* = 7.4 Hz, 2H), 7.76 (d, *J* = 8.0 Hz, 2H), 4.10 (s, 4H), 3.94 (s, 2H), 2.87 (s, 4H); MS (APCI+, *M+1*) 447 .29.

**Synthesis of 1-(4-chloro-3-(trifluoromethyl)phenyl)-3-(4-hydroxyphenyl)urea (SRS15-35, Table 3, entry 9)**

Following the above general **procedure A** with the 4-aminophenol (30 mg, 0.275 mmol), and 1-chloro-4-isocyanato-2-(trifluoromethyl)benzene (72.9 mg, 0.330 mmol), the crude reaction mixture was purified by column chromatography (dichloromethane: methanol = 20:1) to provide the desired sorafenib analog **(SRS15-35)** (72.0 mg, 0.218 mmol, 79%). ^1^H NMR (400 MHz, DMSO) δ 10.67 (s, 20H), 9.19 (s, 20H), 8.93 (s, 21H), 8.09 (d, *J* = 23.7 Hz, 40H), 7.77 (d, *J* = 8.7 Hz, 20H), 7.51 (d, *J* = 8.8 Hz, 39H), 7.45 – 7.41 (m, 1H), 7.18 (d, *J* = 8.7 Hz, 40H); MS (APCI+, *M+1*) 331.29.

**Synthesis of 1-(3,5-bis(trifluoromethyl)phenyl)-3-(4-hydroxyphenyl)urea (SRS15-36, Table 3, entry 10)**

Following the above general **procedure A** with the 4-aminophenol (30 mg, 0.275 mmol), and 1-isocyanato-3,5-bis(trifluoromethyl)benzene (84.0 mg, 0.330 mmol), the crude reaction mixture was purified by column chromatography (dichloromethane: methanol = 20:1) to provide the desired sorafenib analog **(SRS15-36)** (70.0 mg, 0.197 mmol, 70%). ^1^H NMR (400 MHz, DMSO) δ 10.91 (s, 1H), 9.47 (s, 1H), 9.12 (s, 1H), 8.16 (s, 4H), 7.77 (s, 1H), 7.64 (s, 1H), 7.55 (d, *J* = 8.9 Hz, 2H), 7.22 (d, *J* = 8.9 Hz, 2H); MS (APCI+, *M+1*) 366.10.

**Synthesis of 1-(4-chloro-3-(trifluoromethyl)phenyl)-3-(4-ethylphenyl)urea (SRS15-37, Table 3, entry 11)**

Following the above general **procedure A** with the 4-ethylaniline (30 mg, 0.247 mmol), and 1-chloro-4-isocyanato-2-(trifluoromethyl)benzene (65.8 mg, 0.297 mmol), the crude reaction mixture was purified by column chromatography (dichloromethane: methanol = 100:1) to provide the desired sorafenib analog **(SRS15-37)** (76.0 mg, 0.218 mmol, 90%). ^1^H NMR (400 MHz, DMSO) δ 9.12 (s, 1H), 8.74 (s, 1H), 8.11 (d, *J* = 2.0 Hz, 1H), 7.61 (dd, *J* = 8.9, 5.5 Hz, 2H), 7.37 (d, *J* = 8.4 Hz, 2H), 7.13 (d, *J* = 8.4 Hz, 2H), 2.66 – 2.52 (m, 2H), 1.16 (t, *J* = 7.6 Hz, 3H); MS (APCI+, *M+1*) 342.19.

**Synthesis of 1-(3,5-bis(trifluoromethyl)phenyl)-3-(4-ethylphenyl)urea (SRS15-38, Table 3, entry 12)**

Following the above general **procedure A** with the 4-ethylaniline (30 mg, 0.275 mmol), and 1-isocyanato-3,5-bis(trifluoromethyl)benzene (75.8 mg, 0.297 mmol), the crude reaction mixture was purified by column chromatography (dichloromethane: methanol = 20:1) to provide the desired sorafenib analog **(SRS15-38)** (81.0 mg, 0.215 mmol, 87%). ^1^H NMR (400 MHz, DMSO) δ 9.35 (s, 1H), 8.88 (s, 1H), 8.13 (s, 2H), 7.63 (s, 1H), 7.39 (d, *J* = 8.5 Hz, 2H), 7.14 (d, *J* = 8.3 Hz, 2H), 2.56 (q, *J* = 7.6 Hz, 2H), 1.23 – 1.12 (m, 3H); MS (APCI+, *M+1*) 376.19.

**Synthesis of 1-(3,5-bis(trifluoromethyl)phenyl)-3-(2-fluoro-4-hydroxyphenyl)urea (SRS15-39, Table 3, entry 13)**

Following the above general **procedure A** with the 4-amino-3-fluorophenol (30 mg, 0.235 mmol), and 1-isocyanato-3,5-bis(trifluoromethyl)benzene (72.0 mg, 0.283 mmol), the crude reaction mixture was purified by column chromatography (dichloromethane: methanol = 20:1) to provide the desired sorafenib analog **(SRS15-39)** (64.0 mg, 0.167 mmol, 71%). ^1^H NMR (400 MHz, DMSO) δ 9.71 (s, 1H), 9.54 (s, 1H), 8.36 (s, 1H), 8.11 (s, 2H), 7.58 (dd, *J* = 18.6, 9.4 Hz, 2H), 6.73 – 6.50 (m, 2H); MS (APCI+, *M+1*) 383.19.

**Synthesis of 1-(4-chloro-3-(trifluoromethyl)phenyl)-3-(2-fluoro-4-hydroxyphenyl)urea (SRS15-40, Table 3, entry 14)**

Following the above general **procedure A** with the 4-amino-3-fluorophenol (30 mg, 0.235 mmol), and 1-chloro-4-isocyanato-2-(trifluoromethyl)benzene (72.0 mg, 0.283 mmol), the crude reaction mixture was purified by column chromatography (dichloromethane: methanol = 20:1) to provide the desired sorafenib analog **(SRS15-40)** (62.0 mg, 0.178 mmol, 76%). ^1^H NMR (400 MHz, DMSO) δ 9.67 (s, 1H), 9.31 (s, 1H), 8.25 (s, 1H), 8.10 (s, 1H), 7.62 (d, *J* = 14.3 Hz, 3H), 6.61 (dd, *J* = 20.7, 10.6 Hz, 2H); MS (APCI+, *M+1*) 349.10.

**Synthesis of 4-(4-(3-cyclohexylureido)phenoxy)-*N*-methylpyridine-2-carboxamide (SRS14-35, Table 4, entry 1)**

Following the above general **procedure A** with the 4-(4-aminophenoxy)-*N*-methylpyridine-2-carboxamide (30 mg, 0.123 mmol), and isocyanatocyclohexane (19.0 μg, 0.148 mmol), the crude reaction mixture was purified by column chromatography (dichloromethane: methanol = 20:1) to provide the desired sorafenib analog **(SRS15-40)** (38.0 mg, 0.103 mmol, 84%). ^1^H NMR (400 MHz, DMSO) δ 8.75 (d, *J* = 4.7 Hz, 1H), 8.53 – 8.36 (m, 2H), 7.55 – 7.40 (m, 2H), 7.37 (d, *J* = 2.5 Hz, 1H), 7.15 – 6.99 (m, 3H), 6.11 (d, *J* = 7.8 Hz, 1H), 1.81 (d, *J* = 12.0 Hz, 2H), 1.67 (dd, *J* = 8.8, 3.9 Hz, 2H), 1.55 (d, *J* = 12.1 Hz, 1H), 1.37 – 1.26 (m, 2H), 1.24 – 1.13 (m, 3H); MS (APCI+, *M+1*) 369.32.

**Synthesis of 4-(4-(3-cyclohexylthioureido)phenoxy)-*N*-methylpyridine-2-carboxamide (SRS13-100, Table 4, entry 2)**

Following the above general **procedure A** with the 4-(4-aminophenoxy)-*N*-methylpyridine-2-carboxamide (30 mg, 0.123 mmol), and isothiocyanatocyclohexane (21.0 μg, 0.148 mmol), the crude reaction mixture was purified by column chromatography (dichloromethane: methanol = 20:1) to provide the desired sorafenib analog **(SRS15-40)** (39.0 mg, 0.101 mmol, 82%). ^1^H NMR (400 MHz, DMSO) δ 9.41 (s, 1H), 8.77 (d, *J* = 4.3 Hz, 1H), 8.52 (d, *J* = 5.4 Hz, 1H), 7.74 (d, *J* = 6.9 Hz, 1H), 7.59 (d, *J* = 8.6 Hz, 2H), 7.41 (d, *J* = 2.2 Hz, 1H), 7.24 – 7.03 (m, 3H), 1.93 (d, *J* = 9.5 Hz, 2H), 1.77 – 1.54 (m, 3H), 1.35 – 1.14 (m, 5H); MS (APCI+, *M+1*) 384.19.

**General procedure B (Amide bound formation, Table 5)**

To the 3,5-bis(trifluoromethyl)benzoic acid or the 4-chloro-3-(trifluoromethyl)benzoic acid dissolved in dichloromethane, was added oxalyl chloride (1.5 equiv.) and drops of dimethylformamide at 0 ^o^C. The reaction mixture was stirred at room temperature for 4h then all solvent were removed under vaccuo. The desired 4-chloro-3-(trifluoromethyl)benzoyl chloride and 3,5-bis(trifluoromethyl)benzoyl chloride were used without further purification. To the 4-chloro-3-(trifluoromethyl)benzoyl chloride or the 3,5-bis(trifluoromethyl)benzoyl chloride in dry dichloromethane was added the 4-(4-aminophenoxy)-*N*-methylpyridine-2-carboxamide (1 equiv.) and diisopropylethylamine (DIPEA) (3 equiv.). The mixture was stirred at root temperature for 17 h then the solvent was concentrated under vaccuo. The crude reaction mixture was purified by column chromatography (dichloromethane/methanol) to provide the 4-(4-(3,5-bis-(trifluoromethyl)-phenylamidophenoxy)-*N*-methylpyridine-2-carboxamide **(SRS14-95)** and 4-(4-(4-chloro-3-(trifluoromethyl)phenylamido)phenoxy)-*N*-methylpyridine-2-carboxamide **(SRS15-11)** respectively.

**Synthesis of 4-(4-(3,5-bis(trifluoromethyl)phenylamidophenoxy)-*N*-methylpyridine-2-carboxamide (SRS14-95, Table 5, entry 1)**

Following the above general **procedure B** with the 4-(4-aminophenoxy)-*N*-methylpyridine-2-carboxamide (30 mg, 0.123 mmol), and 3,5-bis(trifluoromethyl)benzoic acid (51.0 mg, 0.185 mmol), the crude reaction mixture was purified by column chromatography (dichloromethane: methanol = 20:1) to provide the desired sorafenib analog **(SRS14-95)** (44.0 mg, 0.091 mmol, 74%). ^1^H NMR (400 MHz, CDCl_3_) δ 9.89 (s, 1H), 8.40 (dd, *J* = 16.7, 11.9 Hz, 3H), 8.12 (d, *J* = 4.2 Hz, 1H), 7.95 (s, 1H), 7.89 – 7.68 (m, 2H), 7.45 (s, 1H), 7.18 – 6.91 (m, 3H), 5.46 – 5.27 (m, 1H), 2.95 (t, *J* = 4.5 Hz, 3H).

MS (APCI+, *M+1*) 483.19.

**Synthesis of 4-(4-(4-chloro-3-(trifluoromethyl)phenylamido)phenoxy)-*N*-methylpyridine-2-carboxamide (SRS15-11, Table 5, entry 2)**

Following the above general **procedure B** with the 4-(4-aminophenoxy)-*N*-methylpyridine-2-carboxamide (30 mg, 0.123 mmol), and 4-chloro-3-(trifluoromethyl)benzoic acid (44.0 mg, 0.185 mmol), the crude reaction mixture was purified by column chromatography (dichloromethane: methanol = 20:1) to provide the desired sorafenib analog **(SRS15-11)** (41.0 mg, 0.092 mmol, 74%). ^1^H NMR (400 MHz, DMSO) δ 10.66 (s, 1H), 8.77 (s, 1H), 8.52 (d, *J* = 5.4 Hz, 1H), 8.41 (s, 1H), 8.28 (d, *J* = 7.4 Hz, 1H), 7.92 (t, *J* = 8.8 Hz, 3H), 7.42 (d, *J* = 1.8 Hz, 1H), 7.26 (d, *J* = 8.7 Hz, 2H), 7.20 – 7.12 (m, 1H), 2.80 (d, *J* = 4.5 Hz, 3H); MS (APCI+, *M+1*) 449.19.

**General procedure C (Sulfonamide synthesis, Table 6)**

To the 3,5-bis(trifluoromethyl)benzene-1-sulfonyl chloride or the 4-chloro-3-(trifluoromethyl)benzene-1-sulfonyl chloride in dry dichloromethane was added the 4-(4-aminophenoxy)-*N*-methylpyridine-2-carboxamide (1 equiv.) and diisopropylethylamine (DIPEA) (3 equiv.). The mixture was stirred at root temperature for 17 h then the solvent was concentrated under vacuum. The crude reaction mixture was purified by column chromatography (dichloromethane/methanol) to provide the 4-(4-(3,5-bis(trifluoromethyl)phenylsulfonamido)-phenoxy)-*N*-methylpyridine-2-carboxamide **(SRS14-96)** and 4-(4-(4-chloro-3-(trifluoromethyl)-phenylsulfonamido)phenoxy)-*N*-methylpyridine-2-carboxamide **(SRS14-97)** respectively.

**Synthesis of 4-(4-(3,5-bis(trifluoromethyl)phenylsulfonamido)-phenoxy)-*N*-methylpyridine-2-carboxamide (SRS14-96, Table 6, entry 1)**

Following the above general procedure C with the 4-(4-aminophenoxy)-*N*-methylpyridine-2-carboxamide (30 mg, 0.123 mmol), 3,5-bis(trifluoromethyl)benzene-1-sulfonyl chloride (57.7 mg, 0.185 mmol) and diisopropylethylamine (DIPEA), the crude reaction mixture was purified by column chromatography (dichloromethane: methanol = 20:1) to provide the desired sorafenib analog (SRS14-96) (51.0 mg, 0.098 mmol, 80%). ^1^H ^1^H NMR (400 MHz, CDCl_3_) δ 8.42 (s, 3H), 8.25 (s, 2H), 8.03 (s, 1H), 7.29 (dd, *J* = 6.0, 2.8 Hz, 1H), 7.18 (dd, *J* = 5.8, 3.0 Hz, 2H), 7.11 – 7.02 (m, 3H), 3.06 (dd, *J* = 6.2, 2.7 Hz, 3H); MS (APCI+, *M+1*) 519.19.

**Synthesis of 4-(4-(4-chloro-3-(trifluoromethyl)phenylamido)phenoxy)-*N*-methylpyridine-2-carboxamide (SRS14-97, Table 6, entry 2)**

Following the above general **procedure C** with the 4-(4-aminophenoxy)-*N*-methylpyridine-2-carboxamide (30 mg, 0.123 mmol), 4-chloro-3-(trifluoromethyl)benzene-1-sulfonyl chloride (51.6 mg, 0.185 mmol) and diisopropylethylamine (DIPEA), the crude reaction mixture was purified by column chromatography (dichloromethane: methanol = 20:1) to provide the desired sorafenib analog **(SRS14-97)** (50.0 mg, 0.102 mmol, 83%). ^1^H NMR (400 MHz, CDCl_3_) δ 8.50 (d, *J* = 5.5 Hz, 1H), 8.28 (s, 2H), 8.11 – 8.03 (m, 3H), 7.82 (dd, *J* = 18.2, 5.3 Hz, 4H), 7.28 (s, 1H), 7.16 – 7.07 (m, 5H), 3.06 (d, *J* = 5.0 Hz, 4H); MS (APCI+, *M+1*) 486.19.

**General procedure D (reductive amination reaction, Table 7)^[[2]](#endnote-2)^**

The 4-(4-aminophenoxy)-*N*-methylpyridine-2-carboxamide (1 equiv) and 3,5-bis(trifluoromethyl)-benzaldehyde (2 equiv) or 4-chloro-3-(trifluoromethyl)benzaldehyde (2 equiv) were dissolved in dichloroethane (DCE) and stirred at room temperature in the presence of molecular sieve (4 Å), sodium triacetoxyborohydride (NaBH(OAc)_3_) (1.6 equiv) and drops of acetic acid (AcOH). The reaction mixture was stirred at room temperature under a nitrogen atmosphere for 17h. The reaction mixture was quenched with aqueous saturated NaHCO_3_, and the product was extracted with EtOAc. The EtOAc extract was dried (MgSO_4_), and the solvent was evaporated. The residue was purified by flash-column chromatography on silica gel to provide the desired 4-(4-(3,5-bis(trifluoromethyl)benzylamino)phenoxy)-*N*-methylpyridine-2-carboxamide **(SRS14-99)** or the 4-(4-(4-chloro-3-(trifluoromethyl)benzylamino)phenoxy)-*N*-methylpyridine-2-carboxamide **(SRS14-98)**.

**Synthesis of 4-(4-(3,5-bis(trifluoromethyl)benzylamino)phenoxy)-*N*-methylpyridine-2-carboxamide (SRS14-99, Table 7, entry 1)**

Following the above general **procedure D** with the 4-(4-aminophenoxy)-*N*-methylpyridine-2-carboxamide (50 mg, 0.205 mmol), 3,5-bis(trifluoromethyl)benzaldehyde (59.0 μg, 0.410 mmol) and diisopropylethylamine (DIPEA), the crude reaction mixture was purified by column chromatography (dichloromethane: methanol = 20:1) to provide the desired sorafenib analog **(SRS14-99)** (64.0 mg, 0.136 mmol, 66%). ^1^H NMR (400 MHz, CDCl_3_) δ 8.60 (d, *J* = 3.0 Hz, 1H), 8.40 (d, *J* = 5.6 Hz, 2H), 8.00 (s, 2H), 7.87 (s, 1H), 7.34 (dd, *J* = 5.0, 2.7 Hz, 2H), 7.17 (dd, *J* = 8.2, 2.5 Hz, 2H), 6.95 – 6.90 (m, 2H), 4.50 (s, 2H), 3.03 – 3.01 (m, 3H); MS (APCI+, *M+1*) 469.19.

**Synthesis of 4-(4-(4-chloro-3-(trifluoromethyl)benzylamino)phenoxy)-*N*-methylpyridine-2-carboxamide (SRS14-98, Table 7, entry 2)**

Following the above general **procedure D** with the 4-(4-aminophenoxy)-*N*-methylpyridine-2-carboxamide (50 mg, 0.205 mmol), 4-chloro-3-(trifluoromethyl)benzaldehyde (68.0 μg, 0.410 mmol) and diisopropylethylamine (DIPEA), the crude reaction mixture was purified by column chromatography (dichloromethane: methanol = 20:1) to provide the desired sorafenib analog **(SRS14-98)** (53.0 mg, 0.121 mmol, 59%). ^1^H NMR (400 MHz, CDCl_3_) δ 8.03 (s, 2H), 7.70 (d, *J* = 28.4 Hz, 3H), 7.52 (s, 1H), 7.36 – 7.25 (m, 2H), 7.15 (d, *J* = 8.4 Hz, 1H), 6.92 (d, *J* = 8.5 Hz, 2H), 6.62 (d, *J* = 8.5 Hz, 1H), 4.33 (d, *J* = 53.7 Hz, 2H), 3.02 (d, *J* = 5.9 Hz, 3H); MS (APCI+, *M+1*) 435.19.

**General synthesis of novel sorafenib analogs depicted in Scheme 2 and 3**

***ArS_N_2 reaction (General procedure E)***

To the 1-fluoro-4-nitrobenzene (1 equiv.) in dry DMF (20 mL) was added K_2_CO_3_ (2 equiv.) and various arylalcohol (1.2 equiv.). The mixture was stirred for 17 h at 70^o^C. The solution was poured into water and the organic layer was extracted three times with ethyl acetate. After drying with anhydrous magnesium sulfate the solvents were removed under vacuum. The residue was purified by flash column chromatography on silica gel to provide the desired nitro-ether derivatives **3** (Scheme 2 and 3).

***Hydrogenolysis (General procedure F)***

The desired nitro-ether derivatives **3** (1 equiv.) were dissolved in MeOH (10 mL) and hydrogenated (H_2_ gas) over 10% Pd(OH)_2_ on charcoal for 17 h at room temperature. The solution was filtered through a pad of celite and volatiles were removed under vacuum. The residue was purified by flash-column chromatography on silica gel to provide the desired amine derivatives **4**.

***Addition of various arylamines to various isocyanates (See General procedure A).***

**Synthesis of ethyl 4-(4-(3-(4-chloro-3-(trifluoromethyl)phenyl)ureido)phenoxy)benzoate (SRS14-98, Scheme 2)**

Following the above general **procedure A** the crude reaction mixture was purified by column chromatography (dichloromethane: methanol = 20:1) to provide the desired sorafenib analog **(SRS15-49)** (97.0 mg, 0.204 mmol, 70%). ^1^H NMR (400 MHz, DMSO) δ 9.18 (s, 1H), 8.93 (s, 1H), 8.12 (s, 1H), 7.95 (d, *J* = 8.3 Hz, 2H), 7.59 (dd, *J* = 34.5, 9.1 Hz, 4H), 7.13 – 6.94 (m, 3H), 4.29 (dd, *J* = 12.4, 5.4 Hz, 2H), 1.31 (dd, *J* = 8.9, 5.0 Hz, 3H); MS (APCI+, *M+1*) 479.01.

**Synthesis of ethyl 4-(4-(3-(3,5-bis(trifluoromethyl)phenyl)ureido)phenoxy)benzoate (SRS15-52, Scheme 2)**

Following the above general **procedure A** the crude reaction mixture was purified by column chromatography (dichloromethane: methanol = 20:1) to provide the desired sorafenib analog **(SRS15-52)** (103.0 mg, 0.201 mmol, 69%).^1^H NMR (400 MHz, DMSO) δ 9.23 (d, *J* = 135.0 Hz, 2H), 8.15 (s, 2H), 7.95 (d, *J* = 8.7 Hz, 2H), 7.66 – 7.47 (m, 3H), 7.09 (d, *J* = 8.8 Hz, 2H), 7.01 (d, *J* = 8.7 Hz, 2H), 4.29 (q, *J* = 7.0 Hz, 2H), 1.30 (t, *J* = 7.1 Hz, 3H); MS (APCI+, *M+1*) 513.01.

**Synthesis of 4-(4-(3-(3,5-bis(trifluoromethyl)phenyl)ureido)phenoxy)benzoic acid (SRS15-53, Scheme 2)**

Following the above general **procedure A** the crude reaction mixture was purified by column chromatography (dichloromethane: methanol = 10:1) to provide the desired sorafenib analog **(SRS15-53)** (18.0 mg, 0.038 mmol, 65%). ^1^H NMR (400 MHz, DMSO) δ 12.59 (s, 1H), 9.48 (s, 1H), 9.11 (s, 1H), 8.15 (s, 2H), 7.94 (d, *J* = 8.3 Hz, 2H), 7.68 – 7.45 (m, 3H), 7.10 (d, *J* = 8.3 Hz, 2H), 7.00 (d, *J* = 8.3 Hz, 2H); MS (APCI+, *M+1*) 485.11.

**Synthesis of 4-(4-(3-(4-chloro-3-(trifluoromethyl)phenyl)ureido)phenoxy)benzoic acid (SRS15-54, Scheme 2)**

Following the above general **procedure A** the crude reaction mixture was purified by column chromatography (dichloromethane: methanol = 10:1) to provide the desired sorafenib analog **(SRS15-54)** (17.0 mg, 0.038 mmol, 61%). ^1^H NMR (400 MHz, DMSO) δ 12.59 (s, 1H), 9.48 (s, 1H), 9.11 (s, 1H), 8.15 (s, 2H), 7.94 (d, *J* = 8.3 Hz, 2H), 7.68 – 7.45 (m, 3H), 7.10 (d, *J* = 8.3 Hz, 2H), 7.00 (d, *J* = 8.3 Hz, 2H); MS (APCI+, *M+1*) 451.19.

**Synthesis of 1-(4-chloro-3-(trifluoromethyl)phenyl)-3-(4-(3,4-dimethoxyphenoxy)phenyl)-urea (SRS15-96, Scheme 2)**

Following the above general **procedure A** the crude reaction mixture was purified by column chromatography (dichloromethane: methanol = 20:1) to provide the desired sorafenib analog **(SRS15-96)** (38.0 mg, 0.08 mmol, 50%). ^1^H NMR (400 MHz, DMSO) δ 9.16 (s, 1H), 8.82 (s, 1H), 8.11 (d, *J* = 2.3 Hz, 1H), 7.63 (dt, *J* = 17.7, 5.5 Hz, 2H), 7.44 (d, *J* = 8.9 Hz, 2H), 6.93 (dd, *J* = 8.9, 3.0 Hz, 3H), 6.72 (d, *J* = 2.7 Hz, 1H), 6.47 (dd, *J* = 8.7, 2.7 Hz, 1H), 3.73 (d, *J* = 2.4 Hz, 6H); MS (APCI+, *M+1*) 467.21.

**Synthesis of 1-(3,5-bis(trifluoromethyl)phenyl)-3-(4-(3,4-dimethoxyphenoxy)-phenyl)urea (SRS15-97, Scheme 2)**

Following the above general **procedure A** the crude reaction mixture was purified by column chromatography (dichloromethane: methanol = 20:1) to provide the desired sorafenib analog **(SRS15-97)** (48.0 mg, 0.096 mmol, 59%). ^1^H NMR (400 MHz, DMSO) δ 9.37 (s, 1H), 8.94 (s, 1H), 8.14 (s, 2H), 7.61 (s, 1H), 7.47 (d, *J* = 8.9 Hz, 3H), 6.93 (dd, *J* = 8.7, 5.8 Hz, 4H), 6.72 (d, *J* = 2.6 Hz, 1H), 6.48 (dd, *J* = 8.7, 2.7 Hz, 1H), 3.74 (s, 6H); MS (APCI+, *M+1*) 500.99.

**Synthesis of 1-(4-(3-(aminomethyl)phenoxy)phenyl)-3-(4-chloro-3-(trifluoromethyl)-phenyl)urea (SRN1-16, Scheme 2)**

Following the above general **procedure A** the crude reaction mixture was purified by column chromatography (dichloromethane: methanol = 40:1) to provide the desired sorafenib analog **(SRN1-16)** (10.0 mg, 0.023 mmol, 19%). ^1^H NMR (400 Mz, DMSO-d_6_) δ 9.43 (1H, s), 9.22-8.98 (1H, t), 8.14-8.13 (1H, d), 8.04-7.97 (1H, d), 7.68-7.66 (1H, q), 7.63-7.60 (1H, q), 7.51-7.49 (2H, d), 7.33-7.29 (1H, t), 7.09-7.07 (1H, d), 7.00-6.99 (3H, d), 6.83-6.80 (1H, q), 3.74 (2H, s); MS (APCI+, *M+1*) 436.02.

**Synthesis of 1-(4-(3-(aminomethyl)phenoxy)phenyl)-3-(3,5-bis(trifluoromethyl)phenyl)urea (SRN1-25, Scheme 2)**

Following the above general **procedure A** the crude reaction mixture was purified by column chromatography (dichloromethane: methanol = 40:1 to 5:1) to provide the desired sorafenib analog **(SRN1-25)** (13.0 mg, 0.028 mmol, 25%). ^1^H NMR (400 Mz, DMSO-d_6_) δ 9.94 (1H, s), 9.39 (1H, s), 8.16 (2H, s), 7.66 (1H, s), 7. 56-7 .53 (2H, d) 7.45-7.41 (1H, t) 7.43 (1H, s), 7.21-7.19 (1H, d), 7.14 (1H, s), 7.05-7.02 (2H, d), 7.01-6.98 (1H, q); MS (APCI+, *M+1*) 470.12.

**Synthesis of 1-(4-chloro-3-(trifluoromethyl)phenyl)-3-(4-(3,4-dicyanophenoxy)-phenyl)urea (SRN1-33, Scheme 2)**

Following the above general **procedure A** the crude reaction mixture was purified by preparatory TLC chromatography (dichloromethane: methanol = 100:1) to provide the desired sorafenib analog **(SRN1-33)** (12.0 mg, 0.026 mmol, 31%). ^1^H NMR (400 Mz, DMSO-d_6_) δ 9.32 (1H, s), 9.12 (1H, s), 8.13-8.12 (1H, d), 8.10 (1H, s), 7.77-7.76 (1H, d), 7.70-7.67 (1H, d), 7.65 (1H, s), 7.62 (1H, s), 7.60 (1H, s), 7.40-7.36 (1H, q), 7.18-7.16 (2H, d); MS (APCI+, *M+1*) 457.12.

**Synthesis of 1-(3,5-bis(trifluoromethyl)phenyl)-3-(4-(3,4-dicyanophenoxy)phenyl)urea (SRN1-34, Scheme 2)**

Following the above general **procedure A** the crude reaction mixture was purified by preparatory TLC chromatography (dichloromethane: methanol = 100:1) to provide the desired sorafenib analog **(SRN1-34)** (22.0 mg, 0.045 mmol, 52%). ^1^H NMR (400 Mz, DMSO-d_6_) δ 9.60 (1H, s), 9.38 (1H, s), 8.17 (2H, s), 8.12-8.10 (1H, d), 7.78-7.77 (1H, d), 7.66 (1H, s), 7.64-7.61 (2H, d), 7.39-7.37 (1H, q), 7.19-7.17 (2H, d); MS (APCI+, *M+1*) 457.12.

**Synthesis of 4-(4-(3-(3,5-bis(trifluoromethyl)phenyl)ureido)phenoxy)phenylboronic acid (EJ1-36, Scheme 2)**

(4-(4-aminophenoxy)phenyl)boronic acid (10mg, 0.0386 mmol ) was placed in a vial. Dichloroethane (3mL) was added to the vial. 1-isocyanato-3,5-bis(trifluoromethyl)-benzene (14 uL, 2eq) was then added to the solution. The solution was stirred at 70°C for 17 hours. After 17 hours, the solvent was evaporated under vaccuo. A minimal amount of dichloromethane was added to the mixture, and was purified by using a TLC preparatory plate with one run in 100:1 DCM: Methanol to provide the desired product (***EJ1-36***) (4-(4-(3-(3,5-bis(trifluoromethyl)phenyl)ureido)phenoxy)phenyl)boronic acid (16.95mg, 0.035mmol, 91%) as a dark yellow oil; ^1^H-NMR(CDCl_3_,400MHz,ppm).933 (s, 2H), δ7.576 (s, 1H), δ7.041 (s, 2H), δ7.866-6.821 (m, 2H), δ 6.628-6.517 (m, 3H), δ 6.027 (s, 1H); MS (APCI+, *M+1*) 484.68.

**Synthesis of 4-(4-(3-(4-chloro-3-(trifluoromethyl)phenyl)ureido)phenoxy)phenylboronic acid (EJ1-61, Scheme 2)**

(4-(4-aminophenoxy)phenyl)boronic acid (8mg, 0.349mmol ) was placed in a vial. Dichloromethane (3mL) was added to the vial. 1-chloro-4-isocyanato-2-(trifluoromethyl)benzene (11.56mg, 0.0523 mmol) was then added to the solution. The solution was stirred at room temperature for 17 hours. After 17 hours, the solution was a light yellow color. The stir-bar was removed from the vial and the mixture was rotovapped to give a dark brown oil. A minimal amount of dichloromethane was added to the mixture, and was purified by using a TLC preparatory plate with one run in 100:1 DCM:Methanol, to provide the desired product (***EJ1-61***) (4-(4-(3-(4-chloro-3-(trifluoromethyl)phenyl)ureido)phenoxy)phenyl)boronic acid (12.3mg, 0.273 mmol, 78%) as a dark yellow oil. ^1^H-NMR(CDCl3,400MHz, ppm) δ7.66-7.56 (d, 1H), δ 7.61-7.54 (m, 1H), δ7.42-7.40 (d, 1H), δ7.27-7.25 (m, 4H), δ 6.99-6.97 (m, 2H), δ6.96 (s, 1H), δ6.89-6.86 (d, 1H), δ 6.80 (s, 1H), 6.67-6.65 (d, 1H), 6.52-6.49 ((d,d), 1H); LCMS: m/z (M+) 451,23.

**Synthesis of 1-(4-chloro-3-(trifluoromethyl)phenyl)-3-(4-(pyridin-3-yloxy)phenyl)urea (SRS15-94, Scheme 3)**

Following the above general **procedure A** the crude reaction mixture was purified by column chromatography (dichloromethane: methanol = 40:1) to provide the desired sorafenib analog (**SRS15-94**) (70.0 mg, 0.172 mmol, 80%). ^1^H NMR (400 MHz, DMSO) δ 9.15 (s, 1H), 8.88 (s, 1H), 8.45 – 8.24 (m, 2H), 8.11 (s, 1H), 7.62 (dd, *J* = 27.2, 8.3 Hz, 2H), 7.57 – 7.14 (m, 4H), 7.06 (d, *J* = 8.6 Hz, 2H); MS (APCI+, *M+1*) 408.12.

**Synthesis of 1-(3,5-bis(trifluoromethyl)phenyl)-3-(4-(pyridin-3-yloxy)phenyl)urea (SRS15-95, Scheme 3)**

Following the above general **procedure A** the crude reaction mixture was purified by column chromatography (dichloromethane: methanol = 40:1) to provide the desired sorafenib analog (**SRS15-95**) (71.0 mg, 0.161 mmol, 75%). ^1^H NMR (400 MHz, DMSO) δ 9.15 (s, 1H), 8.88 (s, 1H), 8.45 – 8.11 (m, 3H), 7.62 (m, 2H), 7.57 – 7.14 (m, 4H), 7.06 (d, *J* = 8.6 Hz, 2H); MS (APCI+, *M+1*) 442.32.

**Synthesis of 1-(4-chloro-3-(trifluoromethyl)phenyl)-3-(4-(2-methylbenzo[*d*]thiazol-5-yloxy)phenyl)urea (SRS15-98, Scheme 3)**

Following the above general procedure A the crude reaction mixture was purified by column chromatography (dichloromethane: methanol = 20:1) to provide the desired sorafenib analog **(SRS15-98)** (17.0 mg, 0.036 mmol, 36%). 1H NMR (400 MHz, DMSO) δ 9.17 (s, 1H), 8.89 (s, 1H), 8.11 (d, J = 6.7 Hz, 3H), 7.88 (d, J = 8.8 Hz, 1H), 7.50 (d, J = 8.9 Hz, 2H), 7.17 – 7.10 (m, 2H), 7.03 (d, J = 9.0 Hz, 2H), 2.77 (s, 3H); MS (APCI+, M+1) 478.01.

**Synthesis of 1-(3,5-bis(trifluoromethyl)phenyl)-3-(4-(2-methylbenzo[*d*]thiazol-5-yloxy)phenyl)urea (SRS15-99, Scheme 3)**

Following the above general **procedure A** the crude reaction mixture was purified by column chromatography (dichloromethane: methanol = 20:1) to provide the desired sorafenib analog (**SRS15-99**) (20.0 mg, 0.039 mmol, 40%). 1H NMR (400 MHz, DMSO) δ 9.20 (s, 1H), 8.91 (s, 1H), 8.10-7.88 (d, J = 8.8 Hz, 4H), 7.43-7.10 (m, 4H), 7.03 (m, 2H), 2.77 (s, 3H); MS (APCI+, M+1) 512.31.

**Synthesis of 1-(4-chloro-3-(trifluoromethyl)phenyl)-3-(4-(pyridin-4-yloxy)phenyl)-urea (SRS16-01, Scheme 3)**

Following the above general procedure A the crude reaction mixture was purified by column chromatography (dichloromethane: methanol = 20:1) to provide the desired sorafenib analog (**SRS16-01**) (70.0 mg, 0.172 mmol, 78%). 1H NMR (400 MHz, DMSO) δ 9.35 (s, 1H), 9.19 (s, 1H), 8.12 (s, 1H), 7.95 (d, J = 5.6 Hz, 2H), 7.64 (dd, J = 9.1, 6.7 Hz, 4H), 7.48 (d, J = 6.8 Hz, 2H), 6.27 – 6.16 (m, 2H); MS (APCI+, M+1) 408.03.

**Synthesis of 1-(3,5-bis(trifluoromethyl)phenyl)-3-(4-(pyridin-4-yloxy)phenyl)urea (SRS16-02, Scheme 3)**

Following the above general procedure A the crude reaction mixture was purified by column chromatography (dichloromethane: methanol = 20:1) to provide the desired sorafenib analog (**SRS16-02**) (68.0 mg, 0.155 mmol, 95%). ^1^H NMR (400 MHz, DMSO) δ 10.30 (s, 1H), 9.83 (s, 1H), 8.13 (s, 2H), 7.96 (d, *J* = 7.0 Hz, 2H), 7.67 – 7.55 (m, 3H), 7.49 (d, *J* = 8.2 Hz, 2H), 6.24 (d, *J* = 6.8 Hz, 2H); MS (APCI+, M+1) 442.43.

**Synthesis of 1-(4-chloro-3-(trifluoromethyl)phenyl)-3-(4-(quinolin-8-yloxy)phenyl)urea (EJ1-30, Scheme 3)**

4-(quinolin-8-yloxy)aniline (50 mg, 0.212mmol ) was placed in a vial. Dicholoromethane (3mL) was added to the vial. 1-chloro-4-isocyanato-2-(trifluoromethyl)benzene (70.41 mg, 0.318 mmol) was then added to the solution. The solution was stirred at room temperature for 17 hours. After 17 hours, the solution had a light pink precipitate in it. The solventwas evaporated under vacuum. A minimal amount of dichloromethane was added to the mixture, and was purified by using a TLC preparatory plate with two runs in 100:1 DCM:Methanol, to provide the desired product (***EJ1-30***) 1-(4-chloro-3-(trifluoromethyl)phenyl)-3-(4-(quinolin-8-yloxy)phenyl)urea (57.78 mg, 0.126 mmol, 67%) as very light pink powder. ^1^H-NMR(CDCl3,400MHz,ppm) δ 8.94-8.93 (q, 1H), δ 8.63 (s, 1H), δ 8.35-8.32 (q, 1H), δ 7.91 (s, 1H), δ 7.68-7.67 (m, 2H), δ 7.57-7.46 (m, 3H), δ 7.21-7.19 (q, 1H), δ 7.19-7.07 (d, 2H), δ 6.87-6.84 (d, 2H); MS (APCI+, M+1) 457.89

**Synthesis of 1-(3,5-bis(trifluoromethyl)phenyl)-3-(4-(quinolin-8-yloxy)phenyl)urea (EJ1-33, Scheme 3)**

4-(quinolin-8-yloxy)aniline (40mg, 0.169mmol ) was placed in a vial. Dichloromethane(3mL) was added to the vial. 1-isocyanato-3,5-bis(trifluoromethyl)benzene (58.59 uL, 0.339mmol) was then added to the solution. The solution was stirred at 70°C for 17 hours under reflux. After 17 hours, the solution contained a light pink precipitate. The solvent was evaporated under vacuum. A minimal amount of dichloromethane was added to the mixture, and was purified by using a TLC preparatory plate with two runs in 100:1 DCM: Methanol, to provide the desired product (***EJ1-33***) 1-(3,5-bis(trifluoromethyl)phenyl)-3-(4-(quinolin-8-yloxy)phenyl)urea (51.8mg, 0.105 mmol, 62%) as a medium pink powder. ^1^H-NMR(CDCl3,400MHz,ppm) δ 9.1 (s, 1H), δ 9.934-8.919 ((d,d), 1H), δ8.346-8.325 ((d,d), 1H), δ8.321 (s, 1H), δ 7.660-7.638 ((d,d), 1H), δ 7.574-7.554 (q, 1H), δ 7.505-7.465 (t, 1H), δ 7.419 (s, 1H), δ7.293 (s, 1H), 7.136-7.106 (m, 3H), δ6.876-6.854 (d, 2H);MS (APCI+, M+1) 492.09

**Synthesis of 1-(3,5-bis(trifluoromethyl)phenyl)-3-(4-(5-chloroquinolin-8-yloxy)phenyl)urea (EJ1-35, Scheme 3)**

4-((5-chloroquinolin-8-yl)oxy)aniline (30 mg, 0.111mmol ) was placed in a vial. Dichloroethane (3mL) was added to the vial. 1-isocyanato-3,5-bis(trifluoromethyl)benzene (33uL, 2eq) was then added to the solution. The solution was stirred at 70°C for 17 hours. After 17 hours, the solution contained a pink precipitate. The solvent was evaporated under vacuum. A minimal amount of dichloromethane was added to the mixture, and was purified by using a TLC preparatory plate with two runs in 100:1 DCM:Methanol, to provide the desired product (***EJ1-35***) 1-(3,5-bis(trifluoromethyl)phenyl)-3-(4-((5-chloroquinolin-8-yl)oxy)phenyl)urea (45.4mg, 0.086mmol, 77.7%) as a pink powder; ^1^H-NMR(CDCl3,400MHz,ppm) δ 9.014-9.004 (d, 1H), δ 8.743-8.722 (d, 1H), δ 8.381 (s, 1H), δ 7.712-7.681 (m, 2H), δ 7.606-7.585 (d, 1H), δ 7.495 (s, 1H), δ 7.182-7.123 (d, 2H), δ 7.123-7.103 (d, 1H), δ 6.942-6.921 (d, 2H); MS (APCI+, M+1) 536.20

**Synthesis of 1-(3,5-bis(trifluoromethyl)phenyl)-3-(4-(1,2,3,4-tetrahydroquinolin-8-yloxy)phenyl)urea (EJ1-38, Scheme 3)**

1-(3,5-bis(trifluoromethyl)phenyl)-3-(4-((1,2,3,4-tetrahydroquinolin-8-yl)oxy)phenyl)urea (30 mg, 0.0611 mmol) was placed in a round bottom flask. Ethyl Acetate (4mL) and methanol (1mL) was added to the flask. Palladium hydroxide on charcoal (6.11mg, 10%eq) was added to the solution and a balloon filled with hydrogen gas was fitted to the flask. The solution was stirred at room temperature for 17 hours. The palladium hydroxide was filtered out using celite and the celite was washed with extra ethyl acetate and methanol. The solution mixture was a light pink color. The solvent was evaporated under vacuum to give a dark pink oil. A minimal amount of dicholoromethane was added to the mixture and the desired product was purified by a TLC preparatory plate with one run in 70:1 DCM:Methanol to provide the desired product (***EJ1-38***) 1-(3,5-bis(trifluoromethyl)phenyl)-3-(4-((1,2,3,4-tetrahydroquinolin-8-yl)oxy)phenyl)urea (25.44mg, 0.0514 mmol, 84%) as a dark yellow oil; ^1^H-NMR(CDCl_3_, 400MHz,ppm) δ 9.89 (s, 1H), δ 9.03 (s, 1H), δ 8.05 (s, 2H), δ 7.47-7.41 (d, 3H), δ 6.98-6.91 (d, 2H), δ 6.79-6.73 (d, 1H), δ 6.67-6.60 (d, 1H), δ 6.57-6.50 (t, 1H), δ 3.04-2.94 (q, 3H), δ 2.88-2.84 (t, 2H), 2.02-1.97 (q, 2H); MS (APCI+, M+1) 496.00

**Synthesis of 1-(4-(quinolin-8-yloxy)phenyl)-3-(4-(trifluoromethoxy)phenyl)urea**

**(EJ1-79, Scheme 4)**

4-(quinolin-8-yloxy)aniline (50mg, 0.211mmol ) was dissolved in dichloroethane (3mL). 1-isocyanato-4-(trifluoromethoxy)benzene (48uL, 1.5 eq) was then added to the solution. The solution was stirred at 70 °C for 17 hours. After 17 hours, the solution had produced a light pink precipitate. The sovent was evaporated under vacuum. A minimal amount of dichloromethane was added to the mixture, and was purified by using a TLC preparatory plate with two runs in 40:1 DCM:Methanol to provide the desired product (**1 *EJ1-79***) 1-(4-(quinolin-8-yloxy)phenyl)-3-(4-(trifluoromethoxy)phenyl)urea (5.33 mg, 0.012 mmol, 5.8%) as yellow powder. ^1^H NMR (400 MHz, CDCl_3_) δ 8.97 (d, *J* = 3.1 Hz, 1H), 8.29 (d, *J* = 7.6 Hz, 1H), 7.65 (d, *J* = 8.1 Hz, 1H), 7.57 – 7.47 (m, 2H), 7.40 (d, *J* = 7.8 Hz, 3H), 7.22 (dd, *J* = 15.0, 7.5 Hz, 3H), 7.13 (d, *J* = 7.6 Hz, 2H), 6.98 (d, *J* = 7.8 Hz, 2H); MS (APCI+, M+1) 441.10

***Synthesis of 1-(4-((5-chloroquinolin-8-yl)oxy)phenyl)-3-(4-(trifluoromethoxy)-phenyl)urea* (EJ1-80, Scheme 4)**

4-((5-chloroquinolin-8-yl)oxy)aniline (50mg, 0.185mmol ) was placed in a vial. Dichloroethane(3mL) was added to the vial. 1-isocyanato-4-(trifluoromethoxy)benzene (42uL, 0.28 mmol) was then added to the solution. The solution was stirred at 70 °C for 17 hours. After 17 hours, the reaction mixture produced a light pink precipitate. The stir-bar was removed from the vial and the mixture was rotovapped. A minimal amount of dichloromethane was added to the mixture, and was purified by using a TLC preparatory plate. With one run in 40:1 DCM:MeOH, the mixture was separated. The desired product was scraped off from the TLC prep plate and was eluted from the silica gel to provide (***EJ1-80***) 1-(4-((5-chloroquinolin-8-yl)oxy)phenyl)-3-(4-(trifluoromethoxy)-phenyl)urea ( 39.87mg, 0.084mmol, 45%) as orange powder. ^1^H NMR (400 MHz, DMSO) δ 9.02 (dd, *J* = 5.1, 3.6 Hz, 2H), 8.95 (d, *J* = 45.2 Hz, 1H), 8.61 (dd, *J* = 8.6, 1.4 Hz, 1H), 7.83 – 7.71 (m, 2H), 7.57 (d, *J* = 9.0 Hz, 2H), 7.48 (d, *J* = 8.9 Hz, 2H), 7.29 (d, *J* = 8.6 Hz, 2H), 7.18 (d, *J* = 8.4 Hz, 1H), 7.02 (d, *J* = 8.9 Hz, 2H); MS (APCI+, M+1) 474.43

***Synthesis of 1-(4-((1,2,3,4-tetrahydroquinolin-8-yl)oxy)phenyl)-3-(4-(trifluoromethoxy)phenyl)urea* (EJ1-82, Scheme 4)**

1-(4-((5-chloroquinolin-8-yl)oxy)phenyl)-3-(4-(trifluoromethoxy)phenyl)urea (15 mg, 0.0335 mmol) was placed in a round bottom flask. Ethyl Acetate (4mL) and methanol (1mL) was added to the flask. Palladium hydroxide on charcoal (31.65mg, 31.6mmol) was added to the solution and a balloon filled with hydrogen gas was fitted to the flask. The solution was stirred at room temperature for 17 hours. The palladium hydroxide was filtered out using celite and the celite was washed with extra ethyl acetate and methanol. The solution mixture was a light pink color. The solvent was evaporated to give dark brown oil. A minimal amount of dicholoromethane was added to the mixture and the desired product was purified by using a TLC preparatory plate. With one run in 40:1 DCM:Methanol the mixture was separated. The desired product was scraped off the TLC Prep plate and was eluted from the silica gel to provide (***EJ1-82***) 1-(4-((1,2,3,4-tetrahydroquinolin-8-yl)oxy)phenyl)-3-(4-(trifluoromethoxy)phenyl)urea (3.25mg, 0.0073mmol, 22%) as a light pink powder. ^1^H NMR (400 MHz, CDCl_3_) δ 7.43 – 7.35 (m, 2H), 7.32 – 7.23 (m, 5H), 7.17 (d, *J* = 8.4 Hz, 2H), 7.02 – 6.95 (m, 2H), 6.83 (d, *J* = 7.4 Hz, 1H), 6.73 (d, *J* = 26.2 Hz, 2H), 6.68 (s, 1H), 6.57 (dd, *J* = 14.8, 7.1 Hz, 2H), 3.38 – 3.30 (m, 2H), 2.84 (t, *J* = 6.4 Hz, 2H), 1.99 (dt, *J* = 11.6, 6.4 Hz, 2H); MS (APCI+, M+1) 444.13

**Synthesis of 1-(4-(benzo[d][1,3]dioxol-5-yloxy)phenyl)-3-(4-(trifluoromethoxy)-phenyl)urea (EJ2-03, Scheme 4)**

4-(benzo[*d*][1,3]dioxol-5-yloxy)aniline (50mg, 0.218mmol ) was dissolved in dichloroethane (3mL). 1-isocyanato-4-(trifluoromethoxy)benzene (40uL, 0.28mmol, 1.2eq) was then added to the solution. The solution was stirred at 70 °C for 17 hours. After 17 hours, the solution was a medium yellow color. The solvent was evaporated. A minimal amount of dichloromethane was added to the mixture, and was purified by using a TLC preparatory plate with one run in 5:2 Hexanes:EtOAc, to provide the desired product (***EJ2-03***) 1-(41-(4-(benzo[*d*][1,3]dioxol-5-yloxy)phenyl)-3-(4-(trifluoromethoxy)phenyl)urea (22.1 mg, 0.0511 mmol, 23%) as a light yellow oil. ^1^H-NMR(CDCl3, 400MHz, ppm) δ 7.55-7.52 (t, 1H), δ 7.48-7.45 (d, 2H), δ 7.44-7.42 (d, 4H), δ 7.38-7.36 (d, 1H), δ 7.22-7.18 (m, 6H), δ 7.16-7.13 (d, 1H), δ 6.97-6.95 (m, 1H), δ 6.05-5.99 (d, 2H); MS (APCI+, M+1) 433.

***Synthesis of 1-(4-(4-(adamantan-1-yl)phenoxy)phenyl)-3-(4-(trifluoromethoxy)phenyl)urea* (*EJ1-91*, Scheme 4)**

4-(4-((3*r*,5*r*,7*r*)-adamantan-1-yl)phenoxy)aniline (55mg, 0.172mmol ) was dissolved in dichloroethane (3mL). 1-isocyanato-4-(trifluoromethoxy)benzene (40uL, 1.5eq) was then added to the solution. The solution was stirred at 70 °C for 17 hours.

The solvent was evaporated under vacuum. A minimal amount of dichloromethane was added to the mixture, and was purified by using a TLC preparatory plate with one run in 5:2 Hexanes:EtOac to provide the desired product (***EJ1-91***) 1-(4-(4-(adamantan-1-yl)phenoxy)phenyl)-3-(4-(trifluoromethoxy)phenyl)urea (23.4mg, 0.0448mmol, 26%) as pale yellow powder. ^1^H NMR (400 MHz, CDCl_3_) δ 7.44 (d, *J* = 8.7 Hz, 7H), 7.29 (s, 2H), 7.20 (d, *J* = 8.8 Hz, 9H), 2.13 (s, 3H), 1.94 (s, 6H), 1.81 (d, *J* = 8.3 Hz, 6H); MS (APCI+, M+1) 523.

***Synthesis of 1-(4-(2-(adamantan-1-yl)ethoxy)phenyl)-3-(4-(trifluoromethoxy)-phenyl)urea* (*EJ2-07*, Scheme 4)**

4-(2-((3*r*,5*r*,7*r*)-adamantan-1-yl)ethoxy)aniline (35mg, 0.129mmol ) was dissolved in dichloroethane (3mL). 1-isocyanato-4-(trifluoromethoxy)benzene (23uL, 1.2 eq) was then added to the solution. The solution was stirred at 70 °C for 17 hours. The solvent was evaporated under vacuum. A minimal amount of dichloromethane was added to the mixture, and was purified by using a TLC preparatory plate with one run in 5:2 Hexanes:EtOAc to provide the desired product (***EJ2-07***) 1-(4-(2-(adamantan-1-yl)ethoxy)phenyl)-3-(4-(trifluoromethoxy)phenyl)urea (47.6mg, 0.100mmol, 78%) as dark brown oil. ^1^H-NMR(CDCl3,400MHz,ppm) δ 7.54-7.42 (m, 5H), δ 7.24-7.18 (m, 4H), δ 1.76-1.60 (m, 13H), δ 1.58-1.57 (d, 3H); MS (APCI+, M+1) 475

***Synthesis of 1-(4-chloro-3-(trifluoromethyl)phenyl)-3-(4-((5-chloroquinolin-8-yl)oxy)phenyl)urea*** **(EJ1-69, Scheme 5)**

4-((5-chloroquinolin-8-yl)oxy)aniline (26.57 mg, 0.098mmol ) was placed in a vial. Dichloroethane (3mL) was added to the vial. 1-chloro-4-isocyanato-2-(trifluoromethyl)-benzene ( 32.7 mg, 1.5eq) was then added to the solution. The solution was stirred at 70 °C for 17 hours. After 17 hours, the solution produced a light pink precipitate. The solvent was evaporated under vacuum. A minimal amount of dichloromethane was added to the mixture, and was purified by using a TLC preparatory plate with one run in 40:1 DCM:MeOH to provide the desired product (**EJ1-69**) 1-(4-chloro-3 (trifluoromethyl)phenyl)-3-(4-((5-chloroquinolin-8-yl)oxy)phenyl)urea (14.8mg, 0.030mmol, 31%) as light yellow powder. ^1^H-NMR(DMSO,400MHz,ppm) δ 9.02 (s, 1H), δ 9.01-9.00 (t, 1H), δ 8.94 (s, 1H), δ 8.63-8.60 (q, 1H), δ 8.65-8.24 (d, 1H), δ 7.81-7.81 (d, 2H), δ 7.68-7.65 (m, 2H), δ 7.48-7.47 (d, 4H), δ 7.19-7.14 (d, 1H), δ 7.02-7.01 (d, 2H); MS (APCI+, M+1) 492.2.

***Synthesis of 1-(4-(benzo[d][1,3]dioxol-5-yloxy)phenyl)-3-(4-chloro-3-(trifluoromethyl)phenyl)urea* (*EJ2-01*, Scheme 5)**

4-(benzo[*d*][1,3]dioxol-5-yloxy)aniline (50mg, 0.218mmol ) was placed in a vial. Dichloroethane (3mL) was added to the vial. 1-chloro-4-isocyanato-2-(trifluoromethyl)benzene (58.03mg, 1.2 eq) was then added to the solution. The solution was stirred at 70 °C for 17 hours. After 17 hours, the solution was a medium yellow color. The solvent was evaporated under vacuum. A minimal amount of dichloromethane was added to the mixture, and was purified by using a TLC preparatory plate with one run in 5:2 Hexanes:EtOAc, to provide the desired product (***EJ2-01***) 1-(4-(benzo[*d*][1,3]dioxol-5-yloxy)phenyl)-3-(4-chloro-3-(trifluoromethyl)phenyl)urea (19.6mg, 0.0435 mmol, 20%) as dark yellow oil.

^1^H-NMR(CDCl3,400MHz,ppm) δ 7.88-7.81 (d, 1H), δ 7.84-7.83 (t, 2H), δ 7.63-7.61 (m, 2H), δ 7.59-7.51 (q, 1H), δ 7.49-7.47 (d, 2H), δ 7.38-7.36 (m, 1H), δ 7.27-7.25 (m, 2H), δ 6.98-6.96 (m, 1H), δ 6.79-6.77 (d, 1H), δ 6.58-6.57 (d, 1H), δ 6.51-6.49 (q, 1H), δ 6.05 (s, 1H), δ 6.00 (s, 1H); MS (APCI+, M+1) 452.10

***Synthesis of 1-(4-(adamantan-1-ylmethoxy)phenyl)-3-(4-chloro-3-(trifluoromethyl)phenyl)urea* (*EJ1-70*, Scheme 5)**

4-(((3*r*,5*r*,7*r*)-adamantan-1-yl)methoxy)aniline (7mg, 0.027mmol ) was dissolved in dichloroethane (3mL). 1-chloro-4-isocyanato-2-(trifluoromethyl)benzene (9.05mg, 0.041mmol) was then added to the solution. The solution was stirred at 70°C for 17 hours. The solvent was evaporated under vacuum. A minimal amount of dichloromethane was added to the mixture, and was purified by using a TLC preparatory plate with one run in1:1 Hexanes:EtOAc to provide the desired product (***EJ1-70***) 1-(4-(adamantan-1-ylmethoxy)phenyl)-3-(4-chloro-3-(trifluoromethyl)phenyl)urea (5mg, 0.010mmol, 38%) as yellow powder. ^1^H-NMR (CDCl3, 400MHz, ppm) δ7.87-7.85 (m, 2H), δ7.49-7.47 (d, 1H), δ7.10-7.18 (d, 2H), δ6.90-6.83 (d, 2H), δ6.73 (s, 1H), δ6.44 (s, 1H), δ3.52 (s, 2H), δ2.21-2.10 (m, 3H), δ1.88-1.65 (m, 12H); MS (APCI+, M+1) 479

***Synthesis of 1-(4-(4-(adamantan-1-yl)phenoxy)phenyl)-3-(4-chloro-3-(trifluoromethyl)phenyl)urea* (*EJ1-89*, Scheme 5)**

4-(4-((3*r*,5*r*,7*r*)-adamantan-1-yl)phenoxy)aniline (55mg, 0.172mmol ) was dissolved in dichloroethane (3mL). 1-chloro-4-isocyanato-2-(trifluoromethyl)benzene (57.28mg, 1.5eq) was then added to the solution. The solution was stirred at 70 °C for 17 hours. The solvent was evaporated under vacuum. A minimal amount of dichloromethane was added to the mixture, and was purified by using a TLC preparatory plate with one run in 5:2 Hexanes:EtOAc to provide the desired product (***EJ1-89*)** 1-(4-(4-(adamantan-1-yl)phenoxy)phenyl)-3-(4-chloro-3-(trifluoromethyl)phenyl)urea (21.1mg, 0.039mmol, 23%) as a pale yellow powder.^1^H-NMR(CDCl3,400MHz,ppm) δ 7.61-7.60 (d, 1H), δ 7.48 (s, 1H), δ7.44-7.41 (m, 1H), δ 7.34-7.31 (m, 3H), δ 7.21-7.19 (m, 3H), δ 6.96-6.94 (m, 4H), δ 2.12-2 (s, 3H), δ 1.92-1.91 (d, 6H), δ 1.84-1.75 (m, 6H); MS (APCI+, M+1) 541

***Synthesis of 1-(4-(benzo[d][1,3]dioxol-5-yloxy)phenyl)-3-(3,5-bis(trifluoromethyl)phenyl)urea* (*EJ2-02*, Scheme 5)**

4-(benzo[*d*][1,3]dioxol-5-yloxy)aniline (50mg, 0.218mmol ) was dissolved in dichloroethane (3mL). 1-isocyanato-3,5-bis(trifluoromethyl)benzene (45uL, 0.30 mmol, 1.2eq) was then added to the solution. The solution was stirred at 70 °C for 17 hours. After 17 hours, the solution was a medium yellow color. The solvent was evaporated under vacuum. A minimal amount of dichloromethane was added to the mixture, and was purified by using a TLC preparatory plate with one run in 5:2 Hexanes:EtOAc, to provide the desired product (***EJ2-02***) 1-(4-(benzo[*d*][1,3]dioxol-5-yloxy)phenyl)-3-(3,5-bis(trifluoromethyl)phenyl)urea (17.9 mg, 0.037mmol, 17%) as a light yellow oil. ^1^H-NMR(CDCl3,400MHz,ppm) δ 7.98 (m, 5H), δ 7.68 (m, 2H), δ 7.42-7.39 (d, 2H), δ 7.22-7.19 (d, 2H), δ 6.82-6.79 (m, 1H), δ 6.73-6.52 (m, 2H), δ 6.07 (s, 2H); MS (APCI+, M+1) 485.

***Synthesis of 1-(4-(adamantan-1-ylmethoxy)phenyl)-3-(3,5-bis(trifluoromethyl)phenyl)urea* (*EJ1-71*, Scheme 5)**

4-(((3*r*,5*r*,7*r*)-adamantan-1-yl)methoxy)aniline (7mg, 0.027mmol ) was was dissolved in dichloroethane (3mL). 1-isocyanato-3,5-bis(trifluoromethyl)benzene (5.9uL, 1.5eq) was then added to the solution. The solution was stirred at 70°C for 17 hours. The solvent was evaporated under vacuum. A minimal amount of dichloromethane was added to the mixture, and was purified by using a TLC preparatory plate. With one run in 1:1 Hexanes:EtOAc to provide the desired product (***EJ1-71***) 1-(4-(adamantan-1-ylmethoxy)phenyl)-3-(3,5-bis(trifluoromethyl)phenyl)urea (4.3mg, 0.008mmol, 31%) as light yellow powder. 1H-NMR(CDCl3,400MHz,ppm) δ7.95-7.88 (d, 6H), δ7.58-7.55 (d, 2H),  δ6.99-6.45 (m, 2H), δ3.51 (s, 2H), δ1.82-1.72 (m, 14H); MS (APCI+, M+1) 513

***Synthesis of 1-(4-(4-(adamantan-1-yl)phenoxy)phenyl)-3-(3,5-bis(trifluoromethyl)phenyl)urea* (*EJ1-90*, Scheme 5)**

4-(4-((3*r*,5*r*,7*r*)-adamantan-1-yl)phenoxy)aniline (55mg, 0.172mmol ) was dissolved in dichloroethane (3mL). 1-isocyanato-3,5-bis(trifluoromethyl)benzene (45uL, 1.5eq was then added to the solution. The solution was stirred at 70 °C for 17 hours. The solvent was evaporated under vacuum. A minimal amount of dichloromethane was added to the mixture, and was purified by using a TLC preparatory plate with one run in 5:2 Hexanes:EtOac to provide the desired product (***EJ1-90*)** 1-(4-(4-(adamantan-1-yl)phenoxy)phenyl)-3-(3,5-bis(trifluoromethyl)phenyl)urea (14.6mg, 0.025mmol, 15%) as pale yellow powder. ^1^H-NMR(CDCl3,400MHz,ppm) δ 8.02-8.00 (d, 2H), δ 7.97 (s, 2H), δ 7.67-7.65 (d, 1H), δ 7.45-7.43 (m, 1H), δ 7.40-7.34 (m, 2H), δ 7.26-7.21 (m, 2H), δ 7.13-7.11 (m, 1H), δ 6.98-9.96 (m, 1H), δ 2.15-2.13 (m, 3H), δ 1.97-1.93 (m, 6H), δ 1.83-1.81 (m, 6H); MS (APCI+, M+1) 575,

***Synthesis of 1-(4-(2-(adamantan-1-yl)ethoxy)phenyl)-3-(4-(trifluoromethoxy)-phenyl)urea* (*EJ2-06*, Scheme 5)**

4-(2-((3*r*,5*r*,7*r*)-adamantan-1-yl)ethoxy)aniline (35mg, 0.129mmol ) was dissolved in dichloroethane (3mL). 1-isocyanato-3,5-bis(trifluoromethyl)benzene (27uL, 1.2eq) was then added to the solution. The solution was stirred at 70 °C for 17 hours. The solvent was evaporated under vacuum. A minimal amount of dichloromethane was added to the mixture, and was purified by using a TLC preparatory plate with one run in 5:2 Hexanes:EtOAc to provide the desired product (***EJ2-06***) 1-(4-(2-(adamantan-1-yl)ethoxy)phenyl)-3-(4-(trifluoromethoxy)phenyl)urea ( 50.0 mg,0.095 mmol, 74%) as light yellow powder. ^1^H-NMR(CDCl3,400MHz,ppm) δ 7.924 (s, 5H), δ 7.90-7.89 (d, 2H), δ7.59 (s, 3H), δ 3.85 (s, 9H), δ 1.78-1.62 (m, 3H), δ 1.57 (s, 5H); MS (APCI+, M+1) 527.

1. Bankston, D.; Dumas, J.; Natero, R.; Riedl, B.; Monahan, M.K.; Sibley, R. A scaleable synthesis of BAY 43-9006: A potent raf kinase inhibitor for the treatment of cancer. *Org. Proc. Res. Dev* **2002**, *6*, 777−781. [↑](#endnote-ref-1)
2. Abdel-Magid, A. F.; Carson, K. G.; Harris, B. D.; Maryanoff, C. A.; Shah, R. D. *J. Org. Chem.* **1996,** *61,* 3849-3862 [↑](#endnote-ref-2)
